# The Levels of DAHP Synthase, the First Enzyme of the Shikimate Pathway, Are Related to Free Aromatic Amino Acids and Glutamine Content in *Nicotiana plumbaginifolia* Cell Cultures

**DOI:** 10.3390/plants12132524

**Published:** 2023-07-01

**Authors:** Giuseppe Forlani, Samuele Giberti, Enrico Doria

**Affiliations:** 1 Laboratory of Plant Physiology and Biochemistry, Department of Life Science and Biotechnology, University of Ferrara, 44121 Ferrara, Italy; 2 Laboratory of Plant Biochemistry, Department of Biology and Biotechnology, University of Pavia, 27100 Pavia, Italy; enrico.doria@unipv.it

**Keywords:** amino acid homeostasis, DAHP synthase, end-product control, glyphosate, nitrogen control

## Abstract

Aromatic amino acid homeostasis was investigated in cell suspension cultures of *Nicotiana plumbaginifolia* and was related to the activity of the first enzyme in aromatic biosynthesis, 3-deoxy-D-*arabino*-heptulosonate-7-phosphate (DAHP) synthase. An inverse relationship was found between the intracellular content of free phenylalanine, tyrosine and tryptophan and enzyme specific activity levels, suggesting the occurrence of end-product control mechanisms. Two DAHP synthase isogenes are present in wild tobacco that showed a different expression pattern during the culture growth cycle. Intracellular levels of aromatic amino acids were increased or decreased by adding the culture medium with phenylalanine, tyrosine and tryptophan, or with sublethal doses of the shikimate pathway inhibitor glyphosate, respectively. As a consequence, enzyme levels varied in the opposite direction. The concomitant exogenous supply of glutamine further reduced enzyme activity in mid-log cells, suggesting induction by both aromatic amino acid depletion and nitrogen starvation.

## 1. Introduction

Flavonoids and other aromatic secondary metabolites are synthesized in plants in response to a plethora of environmental stimuli, ranging from pathogen attack to UV exposure. The first committed step in phenolic metabolism is the enzymatic deamination of phenylalanine to yield cinnamic acid, a reaction catalyzed by phenylalanine-ammonia lyase [PAL, EC 4.3.1.5]. Cinnamate synthesis is believed to represent the limiting step in phenylpropanoid biosynthesis [1], whereas the activities of chalcone isomerase [EC 5.5.1.6] and flavonoid 3′5′-hydroxylase [EC 1.14.13.88] are both essential to address the flux of flavonoid intermediates towards anthocyanin production [2]. However, in order to ensure high carbon flux levels through the phenylpropanoid pathway and feed flavonoid biosynthesis, a corresponding flux increase should be induced in the shikimate pathway, the common route that leads to the biosynthesis of the three aromatic amino acids (AAAs) and all the secondary metabolites that are derived therefrom. The first reaction in the shikimate pathway is the condensation of erythrose-4-phosphate (E4P) with phospho*enol*pyruvate (PEP), catalyzed by 3-deoxy-D-*arabino*-heptulosonate-7-phosphate (DAHP) synthase [EC 4.1.2.15 ⇨ 2.5.1.54]. DAHP synthase genes are in fact induced in response to several abiotic and biotic stress conditions that require the synthesis of phenolic protectants to cope with them [3,4,5]. However, microarray data showed that the transcription factors selected and used to engineer anthocyanin accumulation in crops stimulate the transcription of most of the structural genes involved in the phenylpropanoid branch, but do not have an effect upon those in the seven initial steps from E4P and PEP to chorismic acid, the so-called pre-chorismate pathway [6].

Despite a large number of papers dealing with the characterization of the shikimate pathway, the molecular basis of DAHP synthase regulation in plants is still far from being fully elucidated. In enterobacteria, and in other bacteria as well, three enzyme forms exist, each one feed-back regulated by one of the three AAAs [7,8]. In *Saccharomyces cerevisiae* two isozymes were found that are specifically feed-back inhibited by phenylalanine and tyrosine [9]. None of the plant DAHP synthases are regulated by any of the three final products of the pathway [10]. With the only exception of the post-chorismate intermediate L-arogenate [11], no other physiological inhibitor was identified, suggesting that pathway modulation may occur almost exclusively at the genetic level [5]. Separation of a Co^++^ (or Mg^++^)-dependent and a Mn^++^-stimulated isozyme was reported in early studies, and these were found to be functionally located in the cytosol and in the chloroplast, respectively [12]. However, biochemical characterization showed that the Co^++^-dependent enzyme form has extreme substrate ambiguity and uses dyoses (mainly glycolaldehyde) and tryoses at higher rates than E4P. Its involvement in AAA biosynthesis has been therefore questioned, and other functions in plant metabolism have been hypothesized [13]. Subsequently, two genes coding for DAHP synthase were cloned in each of thale cress, potato, tomato and rice ([14], and the references therein), as well as in tobacco [15]. Because of the presence of putative transit peptides, all of these genes are believed to code for a chloroplast-localized, Mn^++^-stimulated enzyme [5]. Precursor translocation into isolated chloroplasts was indeed demonstrated in potato [16]. Because of the in vitro activation by reduced thioredoxin, one Arabidopsis isoenzyme has been proposed to be regulated by the ferredoxin/thioredoxin redox control system in the chloroplast [17]. However, the physiological relevance of such activation was not investigated further. Experimental evidence showing that one isogene (*SHKB*) is induced by external stimuli, such as wounding and pathogen attack, whereas the other (*SHKA*) remains at steady state levels [14,15,18,19] shed some light on this apparent redundancy and led to the hypothesis that a non-inducible gene may be devoted to AAA production for protein synthesis, whereas an inducible gene may specifically respond to changes in secondary metabolism for the production of phenolic protectants [14].

Mn^++^-stimulated DAHP synthase expression was indeed found to increase in response to a variety of conditions, ranging from elicitor treatment [20] to high light [21] and ozone [15]. Enzyme induction was also found following treatment with millimolar concentrations of the phosphonate herbicide glyphosate [22] that inhibits the shikimate pathway enzyme 5-enol-pyruvyl-shikimate-3-phosphate synthase [EC 2.5.1.19], causing AAA depletion [23,24]. However, the molecular mechanisms of the induced rise in DAHP synthase levels are still undisclosed. Moreover, nitrogen starvation is well known to increase anthocyanin biosynthesis in plants [25,26] through the transcription factor-mediated induction of key-genes in the phenylpropanoid pathway [26,27]. Also, some genes in the pre-chorismate branch respond to nitrogen stress, including DAHP synthase [28]. This seems inconsistent, since the synthesis of aromatic amino acids would require more, rather than less, nitrogen. A possible explanation consists in the subsequent release of ammonia from phenylalanine to fuel the phenylpropanoid branch, which can be rapidly recycled for the synthesis of more phenylalanine [29]. However, in this case also, it is not yet known whether the lack of an inorganic nitrogen source or a particular amino acid is responsible for DAHP synthase induction [4]. Recent data on glyphosate-sensitive and glyphosate-resistant biotypes of *Amaranthus palmeri* showed the increased expression of several genes of the shikimate pathway, including DAHP synthase, following treatment with the herbicide. Such effect was abolished by the exogenous administration of AAAs, but once again, the mechanisms underlying this behavior were not elucidated [30].

To shed some more light on these aspects, we investigated the relationship between free AAA content and DAHP synthase activity in cell suspension cultures of a model plant species, *Nicotiana plumbaginifolia*. Results showed that enzyme levels respond to the intracellular concentration of both AAAs and glutamine.

## 2. Results

### 2.1. Concentration of Free Amino Acids in Wild Tobacco Cells Greatly Varies with the Culture Growth Stage, and Aromatic Amino Acid Contents Are Negatively Correlatde with DAHP Synthase Specific Activity Levels

In order to investigate amino acid homeostasis, their intracellular content was quantified in cells harvested at increasing times after subculturing. Results, presented in Table 1, show that in actively proliferating cultures, the amino acid concentration rapidly drops to basal levels, whereas in cells approaching the early stationary phase, it rises again, with an overall 5-fold increase. For most compounds, however, the percent value with respect to total amino acids does not vary remarkably. Interestingly, a reciprocal fluctuation was, on the contrary, evident in the case of glutamine and glutamate. In cells entering the exponential phase, their concentration was similar. In cells approaching the stationary phase of growth, Gln content largely overcame that of Glu, exceeding a 3:1 ratio. AAAs represented about 10% of free amino acids, with Phe accounting for more than 60% of their total content. Also, Phe and Tyr levels varied significantly with time, whereas those of Trp were always near the detection limit of the analytical method used.

**Figure 1:**
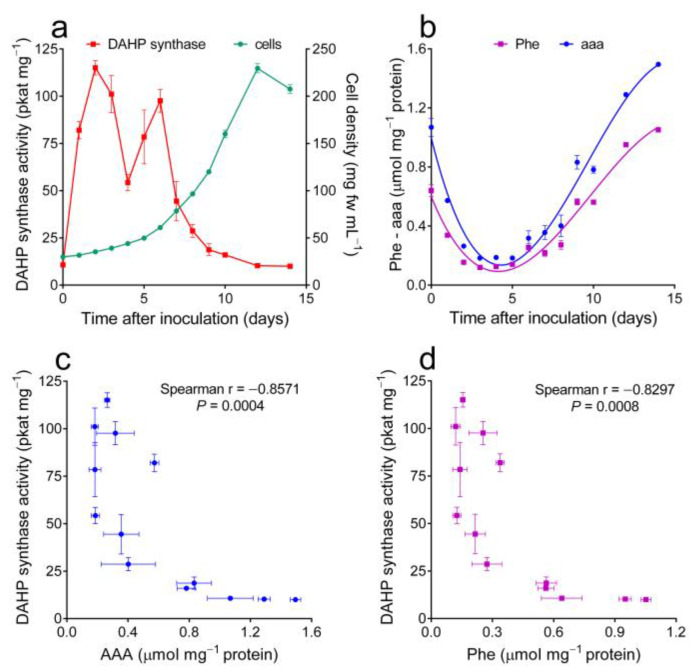
Time course of DAHP synthase specific activity levels and free aromatic amino acid contents during the culture growth cycle of *Nicotiana plumbaginifolia* cells. (**a**) Enzyme levels were quantified in cells harvested at increasing times after the inoculation. The increase in fresh cell biomass is also shown. Data are means ± SEs of six to nine replications obtained in three independent experiments; (**b**) the intracellular concentrations of total AAA and phenylalanine were measured in parallel cultures; (**c**,**d**) results for enzyme and amino acid levels were plotted against each other. Spearman (two-tailed) correlation analysis yielded highly significant results for both AAA and Phe, as indicated.

To measure DAHP synthase activity levels, the analysis of crude extracts was found to be unsuitable. In plant cells, a second protein is indeed present that is able to catalyze the same reaction in vitro, provided that divalent cations, such as Co^++^ or Mg^++^, are added to the reaction mixture [12]. Because of its properties, this Co^++^-dependent enzyme is believed to be involved in other pathways, possibly as a 3-deoxy-D-manno-octulosonate-8- phosphate (KDOP) synthase [31]. In *Pisum sativum*, specific assay conditions were described that allow for activity measurement in crude extracts [10]. Unfortunately, this was not the case with the tobacco enzymes. Following the separation of the Co^++^-dependent and the Mn^++^-stimulated activities via stepwise anion-exchange chromatography, KDOP synthase was the only one to use glycolaldehyde as a substrate (not shown), but with E4P, both activities showed significant catalytic rates even under conditions that were expected to be specific for the other form (Supplementary Figure S1). Therefore, the chromatographic fractionation of crude extracts was routinely required to resolve DAHP synthase from the interfering activity. The activity in the eluate was then related to the amount of protein loaded onto the column, providing an estimate of specific activity levels in the crude extracts. With the adopted protocol, the pattern of DAHP synthase specific activity was followed during the entire growth cycle. Results showed a clear biphasic profile that was found to be highly reproducible (Figure 1a).

Specific activity levels were low in quiescent cells, but after subculturing, a quick and transient increase was evident. However, a second rise was shown thereafter, with the onset of the exponential phase of growth. Later on, even if cells were still actively dividing, enzyme levels rapidly decreased back to barely detectable values. When AAA or Phe contents in parallel cultures (Figure 1b) were plotted against DAHP synthase activity levels (Figure 1c,d), non-linear patterns were found in which high enzyme values corresponded to low free amino acid contents and vice-versa. Statistical analysis showed, in both cases, highly significant correlation coefficients.

### 2.2. Two DAHP Synthase Isogenes Are Expressed in N. plumbaginifolia Cells, and the Corresponding Proteins Can Be Resolved through Anion-Exchange Liquid Chromatography

**Figure 2:**
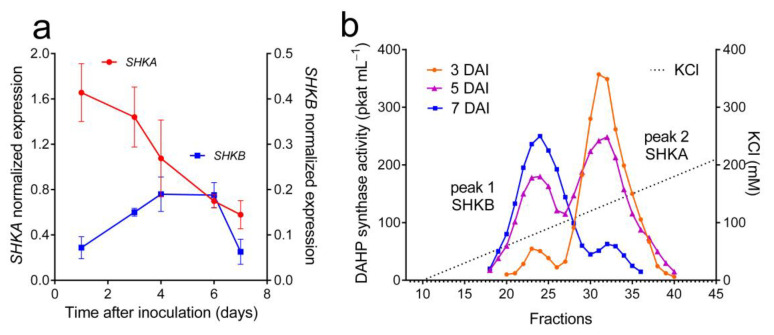
Experimental evidence supporting the expression of two DAHP synthase isoforms in *Nicotiana plumbaginifolia* cell suspension cultures. (**a**) The mRNA isolated from cells harvested at increasing times during the culture growth cycle was retrotranscribed and used for quantitative real-time PCR analysis with primers specific for either isogenes (Table 2). The expression levels were normalized with respect to Actin 7 (accession number FM244697) cDNA. Data are means ± SDs of three independent biological replicates. (**b**) Extracts prepared with cells harvested at a different stage during the growth cycle were fractionated via anion-exchange chromatography, as detailed in the Methods. Results were normalized based on 25 nkat of DAHP synthase activity loaded onto the column.

The biphasic profile of the time course of DAHP synthase specific activity (Figure 1a) would be consistent with the presence of multiple isoenzymes, as described for other plant species. To support this hypothesis, an amplicon-sequencing approach was chosen. Multiple sequence alignment of DAHP synthase isoforms from selected species belonging to *Solanaceae* (Supplementary Figure S2) was used to design primers possibly discriminating the *SHKA* and *SHKB* gene families (Table 2). When mRNA was isolated from *N. plumbaginifolia* cultured cells, retrotranscribed and amplified using these primers, two amplicons of the expected size were obtained. Their sequencing yielded two partial sequences (Supplementary Figure S3) clearly different from each other. In one case, a BLAST analysis indicated 100% amino acid identity with the protein deduced from the *SHKA* sequence from *Nicotiana attenuata* (XP_019228104.1) and 99% with *N. tomentosiformis* (XP_009617912.1) and *N. sylvestris* (XP_009793481.1). Concerning the other amplicon, only 92% identity was found with the SHKB proteins from *N. tabacum* (XP_016476366.1), *N. sylvestris* (XP_009769756.1), *N. tomentosiformis* (XP_009604214.1) and *N. attenuata* (XP_019228823.1), mainly because of the presence of a 20 aa deletion in the carboxy-terminal region (Supplementary Figure S3). Multiple sequence alignment of the two partial proteins with those of other plant DAHP synthases allowed the maximum-likelihood phylogenetic tree, shown in Supplementary Figure S4, to be built. Results clearly showed that DAHP synthases from *N. plumbaginifolia* show higher similarity with homologs from other *Solanaceae* than with each other. On this basis, they were named SHKA and SHKB, respectively, according to the nomenclature used for tobacco [15]. When an expression analysis was carried out by means of quantitative RT-PCR (Figure 2a), with cDNAs prepared from cells harvested at increasing times following inoculation, a remarkably different pattern was obtained: *SHKA* was found to be expressed at a high level in cells resuming proliferation, whereas *SHKB* showed a lower level of transcription, with a maximum at the onset of the exponential phase of growth.

To gain further information, extracts from cells harvested at different times after inoculation were fractionated on an anion-exchange column using a different protocol. Instead of using a step-wise NaCl gradient, proteins were eluted by means of a linear, flat gradient from 0 to 400 mM KCl. Under these conditions, two DAHP synthase peaks were indeed resolved (Figure 2b). The same elution pattern was obtained when purification was performed in the presence of protease inhibitors (1 mM PMSF and 1 μM pepstatin A). Rechromatography of pooled fractions from one peak yielded a single peak in the same position of the chromatogram (not shown). The relative proportion of the two activities varied with the age of the culture. A comparison of these results with the expression pattern depicted in Figure 2a allowed us to speculate on the isoform most likely corresponding to each peak, with SHKA and SHKB showing an inverse order of elution (Figure 2b).

### 2.3. Variations in Intracellular Free Aromatic Amino Acid Pools, Induced by Treatment with Either Exogenous AAA or the Aromatic Synthesis Inhibitor Glyphosate, Cause the Consistent and Opposite Fluctuation of DAHP Synthase Specific Activity

**Figure 3:**
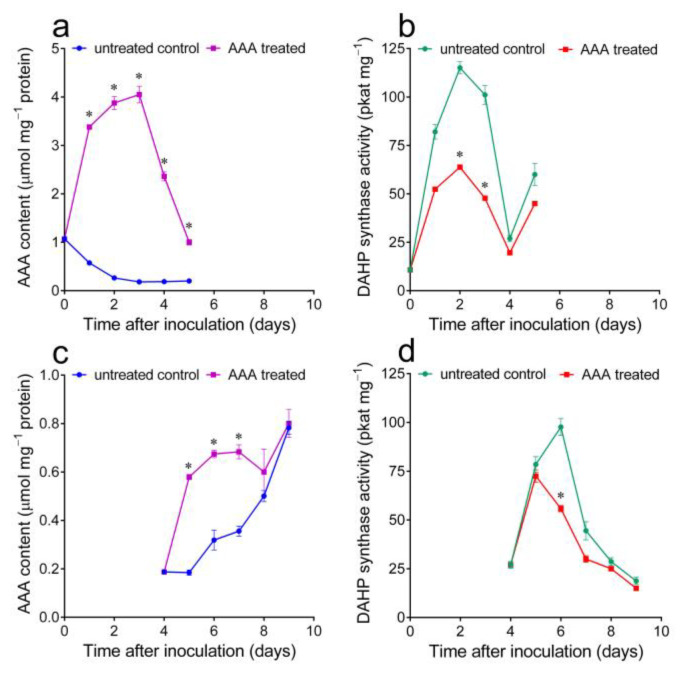
Effect of exogenously supplied AAAs on DAHP synthase specific activity levels in *Nicotiana plumbaginifolia* suspension-cultured cells. (**a**,**c**) Phe (0.5 mM), Tyr (0.25 mM) and Trp (0.125 mM) were added to the culture medium upon subculturing (**a**) or four days after inoculation (**c**), and the resulting intracellular free AAA pools were quantified at increasing times and compared with those in untreated controls. (**b**,**d**) DAHP synthase activity was measured in extracts prepared from the same samples as in (**a**) and (**c**), respectively. In all cases, data are the means ± SEs of three independent biological replicates. The asterisk denotes a value in treated samples that is significantly different from that in untreated controls (*p* < 0.01), as determined using a multiple *t*-test corrected for multiple comparisons using the Holm–Sidak method.

To obtain more evidence supporting a relationship between the free AAA content and DAHP synthase level, an increase in the former was induced by treating cultured cells with exogenously supplied amino acids. The addition of millimolar levels of Phe, Tyr and Trp, both alone or in pairs, was found to severely inhibit growth (data not presented). To avoid this, the highest non-inhibitory concentration of an almost equimolar mixture of the three final products (500 µM Phe, 250 µM Tyr and 125 µM Trp) was added to the culture medium upon subculturing. Under these conditions, a significant increase in the intracellular AAA content was in fact obtained (Figure 3a), well above both the maximal physiological concentrations previously found and the external levels, suggesting the occurrence of active uptake. As a consequence, a significant decrease in DAHP synthase levels was found, although its increase over basal levels was not completely prevented (Figure 3b). The progressive utilization of exogenous AAAs caused a rapid decrease in their intracellular pools back to control levels 3 to 5 days after the treatment, hampering the evaluation of the resulting effect in the second part of the culture growth cycle. The experiment was therefore repeated by adding exogenous AAAs 4 days after subculturing. In this case, most likely because of the higher cell density, their intracellular content increased to lower levels (Figure 3c). Notwithstanding this, a decrease in enzyme levels was again evident (Figure 3d).

**Figure 4:**
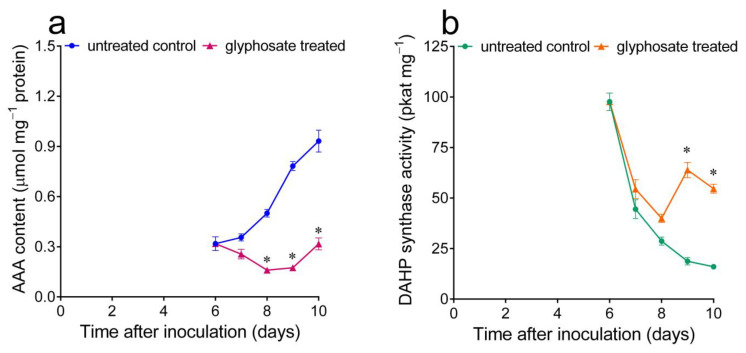
Effect of a sublethal dose of the phosphonate herbicide glyphosate on AAAs and DAHP synthase activity levels in *Nicotiana plumbaginifolia* cultured cells. (**a**) The intracellular free AAA pools were quantified at increasing times after the addition of 0.5 mM glyphosate to the culture medium 6 days after subculturing and compared with those in untreated controls. (**b**) DAHP synthase activity was measured in extracts prepared from the same samples. In all cases, data are the means ± SE of three independent biological replicates. The asterisk represents a value in treated samples that is significantly different from that in untreated controls (*p* < 0.01), as determined using a multiple *t*-test corrected for multiple comparisons using the Holm–Sidak method.

If an inverse correlation exists between free AAAs and DAHP synthase levels, a reduction in the intracellular concentration of the three final products is expected to prevent the decline in enzyme specific activity that takes place in mid-log cultures (Figure 1a). To verify this possibility, cells were treated with the phosphonate herbicide glyphosate, which acts by interfering with the shikimate pathway. The sensitivity of the culture greatly varied with the stage at which glyphosate was added. If an excessive concentration was employed, the consequent inhibition of AAA synthesis quickly led to the rapid accumulation of free Glu, Gln and Ala at the highest levels (not shown), most likely due to a complete block of protein synthesis. With a proper dose (0.5 mM) administered 6 days after the inoculation, only the AAA content was, on the contrary, affected within the subsequent 48–72 h. Under these conditions, a delay in the increase in the intracellular concentration of the three final products was in fact found (Figure 4a). This was reflected by DAHP synthase levels, which remained high until the late exponential phase of growth (Figure 4b).

### 2.4. The Intracellular Concentration of Free Glutamine Also Influences DAHP Synthase Levels in Actively Proliferating Cells

**Figure 5:**
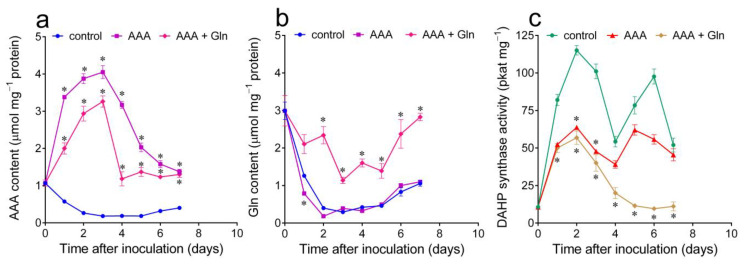
Effect of exogenous AAAs and glutamine, alone or in combination, on free amino acids and DAHP synthase activity levels in *Nicotiana plumbaginifolia* cultured cells. (**a**) The intracellular free AAA pools were quantified at increasing times after the addition of 0.5 mM Phe, 0.25 mM Tyr and 0125 mM Trp, with or without 2.5 mM glutamine, and compared with those in untreated controls. (**b**) The intracellular free glutamine levels were measured in the same samples. (**c**) DAHP synthase activity was measured in extracts prepared from the same samples. In all cases, data are the means ± SE from at least three independent biological replicates. The asterisk indicates a value in treated samples that is significantly different from that in untreated controls (*p* < 0.01), as determined using a multiple *t*-test corrected for multiple comparisons using the Holm–Sidak method.

Following the treatment with exogenous AAAs, DAHP synthase activity was significantly reduced, but not completely abolished (Figure 3b,d), although free amino acid levels were comparable or even higher than those in untreated cells harvested in the late exponential phase of growth, when activity was almost undetectable (Figure 1a). Taking into account that the first enzyme in the shikimate pathway had been reported to be induced by nitrogen starvation [28] and that in untreated cultures, high AAA content was always accompanied by high levels of free glutamine (Table 1), the effect of a combination of exogenous AAAs and glutamine was also investigated. In order to avoid both cell growth inhibition and a rapid return to control levels, low concentrations of AAAs (0.5 mM Phe, 0.25 mM Tyr and 0125 mM Trp) and glutamine (2.5 mM) were added repeatedly to the culture medium, upon subculturing and 3 and 6 days thereafter. As shown in Figure 5a,b, this treatment resulted in high levels of intracellular amino acid pools throughout. Under these conditions, exogenous AAAs caused about a 50% reduction in DAHP synthase specific activity. When glutamine was also added, the same pattern was evident early after inoculation. On the contrary, an additive effect was found at the subsequent onset of the exponential phase of growth, when enzyme activity levels dropped to basal levels (Figure 5c).

## 3. Discussion

Intracellular concentrations of free AAAs and DAHP synthase activity levels were found to significantly vary during the growth cycle of *N. plumbaginifolia* cultured cells, showing an opposite pattern (Figure 1a,b). While feed-back inhibition mechanisms have been proven for enzymes at branching points downstream of chorismic acid [32], despite extensive experimentation, plant DAHP synthase has never been found to be inhibited by physiological concentrations of the three final products [31]. Modulation of the pre-chorismate pathway is therefore believed to occur almost exclusively at the genetic level [5], yet the molecular basis of such regulation had never been investigated in detail. In several instances, treatments causing a reduction in the AAA biosynthetic rate were found to induce DAHP synthase expression. When potato mid-log phase cells were exposed to the shikimate pathway inhibitor glyphosate, enzyme specific activity increases several-fold within 24 h [22]. Gene expression profiling showed a significant increase in DAHP synthase transcription 24 h after treatment with glyphosate in herbicide-sensitive soybean plants, but not in herbicide-tolerant transgenic plants [33]. Analogous results were recently described with glyphosate-sensitive and glyphosate-resistant biotypes of the weed *Amaranthus palmeri* [30]. When the expression of the bifunctional 3-dehydroquinate dehydratase (EC 4.2.1.10)/shikimate dehydrogenase (EC 1.1.1.25) was suppressed in tobacco by RNAi, transgenic lines displayed severe growth retardation and a reduced content of AAAs and downstream products. As a consequence, the accumulation of DAHP synthase transcripts increased up to four times with respect to wild-type control levels, whereas that of other shikimate pathway genes was found to be unaffected [34]. DAHP synthase was also found to be induced early in response to a wide array of (a)biotic stress conditions requiring the synthesis of phenolic protectants [31]. Interestingly, in several studies in which the induction kinetics were investigated, the gene coding for DAHP synthase showed a slightly delayed response compared to that coding for PAL (e.g., [19,35]). The possibility therefore exists that the expression of the first enzyme in the shikimate pathway is triggered by the reduction in free Phe levels caused by increased PAL activity. However, in all these previous papers, the nature of the molecular effector(s) was not investigated in detail. To the best of our knowledge, this is the first study in which targeted variations in AAA content were shown to cause changes in DAHP synthase specific activity levels (Figure 3 and Figure 4). Since the response occurred rapidly after treatment and conditions were selected to minimize unrelated effects, a causal relationship seems likely. The investigation of the effect of a specific variation in Phe alone was not feasible because of the consequent inhibition of cell growth. Only treatment with almost equimolar doses of the three AAAs did not exert an inhibitory effect on cell proliferation. This is consistent with previous reports showing that, if provided alone, AAAs severely affect the growth of plant cell cultures [36,37].

A decrease in free Phe caused by activation of the phenylpropanoid pathway could also be the reason for the induction of DAHP synthase under nitrogen starvation [28], a condition that promotes anthocyanin synthesis [25,26]. As the major form of nitrogen available in the soil, nitrate represents a signal for plant growth and metabolism, but still, little is known about the molecular components that constitute or mediate this signal [38]. Nitrate regulates parts of phenylpropanoid, flavonoid and anthocyanin metabolism *per se*, since nitrate addition to N-starved seedlings lead to changes in gene expression that occur much earlier than any increase in organic N status [39]. In MS medium, nitrogen is provided as both nitrate and ammonia, at concentrations (21 mM NH_4_NO_3_ and 19 mM KNO_3_) high enough to ensure non-limiting conditions. However, data described herein suggest the possible occurrence of a more complex mechanism, in which the nitrogen status of the cell is sensed through the resulting intracellular levels of glutamine (or the glutamine-to-glutamate ratio). As a result, low glutamine levels cause a further increase in DAHP synthase specific activity over that induced by a low AAA content in actively proliferating cells (Figure 5). Since glutamine and glutamate represent the first batch of organic nitrogen compounds synthesized de novo in plants, it is not surprising that they may act as signals triggering regulative responses. Several nitrogen-sensing systems among those elucidated to date in bacteria and animals, such as the PII, GAAC, iGluR and, partially, NIT systems, are mainly responsive to amino acids, and some of these mechanisms indeed seem to also exist in plants [40].

Data described herein support the presence, in cultured cells of wild tobacco, of two Mn^++^-stimulated DAHP synthase isozymes channeling precursors towards the synthesis of aromatic compounds (Figure 2). Results are consistent with an increasing array of data showing that at least two different DAHP synthase genes, both coding for a putative transit peptide, are present in higher plants ([14], and the references therein). The use of specific primers designed on the basis of sequences available for other members of *Solanaceae* yielded two amplicons, of which sequences showed higher similarity to the *SHKA* and *SHKB* gene families than to each other. Three *SHKB* and one *SHKA* genes were reported in tobacco [41], but unlike *N. plumbaginifolia* (2n = 20), tobacco is an allotetraploid species, and only two of those genes showed significant expression levels in planta [15]. Of each pair, *SHKB* was found to be induced by external stimuli [14,15,18,42] and therefore linked to the synthesis of aromatic secondary metabolites, whereas *SHKA* was supposed to produce AAA for protein synthesis [14]. In *N. plumbaginifolia* cultures, SHKA showed maximal levels in cells resuming proliferation and may indeed fulfill the latter role, being expressed when the need for de novo AAA production for protein synthesis is high. Further elements would be, on the contrary, needed to speculate on the involvement of SHKB in the production of phenolic protectants. However, in wild tobacco cells, *SHKB* showed expression levels one order of magnitude lower than those of *SHKA* (Figure 2b), and exogenously supplied glutamine was consistently found to affect DAHP synthase levels in connection with *SHKB* maximal expression (Figure 2 and Figure 5). Chemical/mechanical stress upon subculturing was found to, *per se*, cause anthocyanin synthesis and/or PAL induction in *Petunia hybrida* [43] and *Oryza sativa* [44] suspension cultured cells. As an alternative, as suggested by the results obtained when treating actively proliferating cells with exogenous amino acids (Figure 5), the expression of *SHKB* could be simply connected to low levels of either/both AAAs and glutamine inside the cell.

More experimental evidence will be required to shed light on the exact molecular mechanisms underlying the modulation of DAHP synthase levels. In particular, it is still unclear how the plant monitors its nitrogen status, which compounds are sensed and the identity of the signaling pathways responsible for their detection, with the plastidic PII-dependent pathway, the family of glutamate-like receptors, the target of rapamycin (TOR) signaling pathway and the general control non-derepressible 2 pathway being the most likely candidates [45]. Moreover, whether AAAs and glutamine are components of the same or different signaling transduction pathways needs to be assessed. Finally, the modulation of activity levels could depend on either transcriptional or translational control, as reported in the case of the TOR pathway [46]. Interestingly, a recent study found that 15 proteinogenic amino acids can reactivate TOR when exogenously supplied to nitrogen-starved *Arabidopsis thaliana* seedlings, strengthening the possibility that amino acids are the upstream regulators of TOR-signaling pathways [47]. In any case, the present data clearly show a relationship between the intracellular contents of free AAAs and glutamine and activity levels of the first enzyme in the shikimate pathway. Whatever the transduction mechanism, a better comprehension of these aspects is desirable, which is expected to provide new tools to control the levels of aromatic secondary metabolites in plants [48].

## 4. Materials and Methods

### 4.1. Plant Materials, Growth Conditions and Amino Acid Treatments

Cell suspension cultures of *Nicotiana plumbaginifolia* Viviani were grown in 0.5 L Erlenmeyer flasks in MS medium containing 0.3% (*w*/*v*) sucrose and 0.5 mg L^−1^ of both 2,4-dichlorophenoxyacetic acid and 6-benzylaminopurine. Incubation was under dim light at 24 ± 1 °C on a rotary shaker (100 rpm). Subcultures were generated every 2 weeks by transferring 25 mL aliquots to 100 mL of fresh medium. The effect of either a factorial of various combinations of the three AAAs and glutamine or glyphosate (brought to pH 6.0 with KOH) on exponentially growing cells was measured as described previously [24]. Briefly, cell samples withdrawn from the stock cultures in the late exponential phase of growth were used to inoculate 100 mL culture flasks to a density of 1.0–1.2 mg mL^−1^ (dry weight) in a final volume of 25 mL. Filter-sterilized compounds were added just after the density of the cell population reached 2.0 mg mL^−1^ (dry weight). After a further 8 d of incubation, when untreated controls reached the early stationary phase of growth, cells were harvested by performing vacuum filtration, and the dry weight increase was determined for each sample following oven drying at 90 °C for 48 h.

### 4.2. DAHP Synthase Extraction

Cells were harvested via vacuum filtration on nylon filters (64 µm mesh), washed twice with distilled water, weighed and ground in a mortar with liquid nitrogen to a fine powder, which was suspended in 2 mL g^−1^ of 25 mM Tris-HCl buffer, pH 7.2, containing 0.5 mM dithiothreitol (DTT). Alternatively, small amounts of cells were directly suspended in the same buffer and extracted with a Teflon-in-glass Potter homogenizer using 3 × 8 strokes, taking care to avoid sample heating. All subsequent operations were carried out at 0–4 °C. Following the addition of 10 mg mL^−1^ insoluble polyvinyl-polypyrrolidone to remove phenolics, the homogenate was centrifuged for 15 min at 12,000× *g*. The supernatant was fractionated with ammonium sulphate (70% of saturation). Precipitated proteins were sedimented through centrifugation, as above, and resuspended in a minimal volume of extraction buffer. Extracts were loaded at a constant flow of 1 mL min^−1^ onto a DEAE-Sephacel column (1.5 cm diameter, 10 mL bed volume) equilibrated with extraction buffer. After washing with 20 mL of buffer containing 50 mM NaCl, DAHP synthase was eluted stepwise with 30 mL of buffer containing 150 mM NaCl while collecting 2 mL fractions. Protein content was measured based on the method of [49], using bovine serum albumin as the standard.

### 4.3. DAHP Synthase Assay

DAHP synthase activity was measured as previously described [23,50], with minor modifications. The reaction mixture contained 50 mM EPPS-KOH pH 7.75, 0.5 mM E4P, 2 mM PEP, 0.7 mM MnCl_2_ and the enzyme. Since Mn^++^-stimulated DAHP synthase from higher plants shows hysteretic activation [17], the enzyme was preincubated for 10 min at 35 °C with 1 mM DTT prior to addition to the reaction mixture. After an appropriate incubation period (up to 30 min) at 35 °C, the reaction was stopped by the addition of 25 µL of a 20% (*w*/*v*) solution of TCA. Following centrifugation for 3 min at 14,000× *g*, 100 µL of the supernatant was mixed with the same volume of a 25 mM Na metaperiodate solution in 0.125 N sulphuric acid. DAHP oxidation was allowed to proceed for 30 min at 37 °C, and then, excess periodate was destroyed by the addition of 200 µL of a 2% (*w*/*v*) solution of sodium arsenite in 0.5 N HCl. Each sample was then added to 800 µL of a 0.3% (*w*/*v*) solution of thiobarbituric acid and incubated for 10 min at 100 °C. The consequent increase in absorbance at 549 nm was read against exact blanks inactivated at time 0, and the activity was calculated on the basis of a molar extinction coefficient of 45,000 M^−1^ cm^−1^ [51].

### 4.4. Chromatographic Separation of DAHP Synthase Isoforms

The 0–70% ammonium sulphate-saturated fraction was desalted via passage through a Bio-Gel P6DG (Bio-Rad, Hercules, CA, USA) column equilibrated with extraction buffer. Desalted samples were loaded onto a DEAE-Sephacel column (2.5 cm diameter, 25 mL bed volume) equilibrated with the same buffer. After extensive washing, retained proteins were eluted with a computer-controlled (Data System 450; Kontron, Munchen, Germany) linear gradient from 0 to 400 mM KCl (400 mL), collecting 6 mL fractions.

### 4.5. Amino Acid Extraction, Separation and Quantification

For amino acid analysis, plant material was suspended in 1 mL g^−1^ of a 3% (*w*/*v*) solution of 5-sulphosalicylic acid and extracted with a Teflon-in-glass Potter homogenizer, as described. Following centrifugation for 10 min at 12,000× *g*, 1 mL aliquots of the supernatant were brought to dryness at RT in a centrifugal vacuum concentrator (Eppendorf, Model 5301). Residues were reconstituted with 0.1 mL of 2.5 N NaOH, resulting in a pH value of 10.2 ± 0.2, and immediately analysed. Samples were mixed with the same volume of *o*-phthaldialdehyde solution (0.5 M in 0.5 M sodium borate buffer, pH 10.0, containing 0.5 M β-mercaptoethanol and 10% [*v*/*v*] methanol). After exactly 60 s, 20 µL of derivatized samples was injected onto a 4.6 × 250 mm Zorbax ODS column (Rockland Technologies, Newport, DE, USA) equilibrated with 59% solvent A (50 mM sodium phosphate-50 mM sodium acetate buffer, pH 7.5, containing 2% [*v*/*v*] of both methanol and tetrahydrofuran) and 41% solvent B (65% methanol). Elution proceeded at a flow rate of 60 mL h^−1^ using a computer-controlled (Data System 450; Kontron, Munchen, Germany) complex gradient from 41 to 100% solvent B as previously described [52], monitoring the eluate at 340 nm. This procedure allowed for the complete resolution of equimolar mixtures of derivatizable amino acids (all 20 protein amino acids, except Pro and Cys), with a detection limit of about 0.1 nmol. Peaks were integrated by area, with variation coefficients ranging from 0.8 to 3.2%. Proline and total amino acid contents were quantified using the acid ninhydrin method, as described [53]. Results were expressed as µmol mg^−1^ soluble protein (to allow for a proper comparison with specific activity values, which are also expressed as a function of the soluble protein), of which, the content was determined as described in parallel extracts of cells from the same culture.

### 4.6. PCR Cloning and RT-PCR Analysis

Total RNA was extracted from 50 mg of plant material using the Plant RNA Isolation kit (Agilent Technologies, Santa Clara, CA, USA), according to the manufacturer’s instructions. Following spectrophotometric quantification and assessment of RNA integrity, RNA (1 μg) was incubated at 30 °C for 30 min with 5 U of amplification-grade DNase I (Sigma AMPD1), treated for 10 min at 70 °C to denature both the DNase I and the RNA, chilled on ice and immediately used for single-strand cDNA synthesis via reverse transcription in a 20 μL reaction mixture using the iScript cDNA Synthesis Kit (Bio-Rad). First-strand cDNA was used as the template for PCR amplifications using specific primers for target and housekeeping genes (Table 2).

For end-point PCR analysis, the amplification was carried out in a Mastercycler Personal thermal cycler (Eppendorf) in a 20 μL reaction mixture containing 10 μL iQTMSupermix (Bio-Rad), 20 pmol forward and reverse primers and 10 to 50 ng of cDNA. Cycling conditions consisted of an initial 5 min at 95 °C, followed by 30 s of denaturing at 94 °C, 30 s of annealing at 59 °C and 60 s of elongation at 72 °C, repeated for 28–34 cycles, and with a 5 min final extension at 72 °C. Products were analyzed via gel electrophoresis (1% agarose).

qPCR was carried out with 25 ng of cDNA in a volume of 15 µL on a CFX96 Real-Time PCR Detection System (Bio-Rad) using SsoAdvanced™ Universal SYBR^®^ Green Supermix (Bio-Rad) with the following thermal profile: enzyme activation at 98 °C for 30 s, followed by 40 cycles of denaturation for 5 s at 95 °C and annealing and extension for 15 s at 61 °C. Melting curve analysis was performed after cycle completion to validate the amplicon identity. The expression levels of the housekeeping gene were used to obtain the relative expression of each target gene with the ΔΔ_Ct_ method [54] using the Gene Expression Module of CFX Manager software^TM^ (Version 3.1, Bio-Rad). For each treatment, three biological replications were performed, and two technical replicates were run for each sample in the qPCR analysis.

### 4.7. Statistical Analysis

Presented results are the means ± SDs over at least three replications. Data were analysed by means of standard statistical procedures using the Prism 6 (version 6.03, GraphPad Software, Inc., San Diego, CA, USA) package. Where differences are reported, unless indicated otherwise, they are at the 99% confidence level (*p* < 0.01).

## Figures and Tables

**Table 1:** Free amino acid content in *Nicotiana plumbaginifolia* suspension-cultured cells at different stages during the culture growth cycle (DAI, day after inoculation).

AA	1 DAI		4 DAI		7 DAI		10 DAI	
	µmol (mg prot)^−1^	%	µmol (mg prot)^−1^	%	µmol (mg prot)^−1^	%	µmol (mg prot)^−1^	%
Asp	0.19 ± 0.01	3.5	0.07 ± 0.01	3.6	0.11 ± 0.00	2.7	0.19 ± 0.02	2.0
Glu	0.57 ± 0.01	10.7	0.39 ± 0.06	19.2	0.60 ± 0.05	13.9	0.91 ± 0.13	9.5
Asn	0.21 ± 0.01	4.0	0.03 ± 0.00	1.3	0.09 ± 0.01	2.1	0.23 ± 0.05	2.4
Ser	0.11 ± 0.01	2.1	0.15 ± 0.01	7.5	0.14 ± 0.01	3.3	0.12 ± 0.01	1.3
Gln + His	1.26 ± 0.06	23.8	0.42 ± 0.06	20.6	1.16 ± 0.06	27.0	3.06 ± 0.62	31.7
Arg	0.49 ± 0.01	9.3	0.05 ± 0.01	2.3	0.39 ± 0.03	9.0	0.84 ± 0.20	8.7
Gly	0.22 ± 0.07	4.1	0.03 ± 0.00	1.6	0.11 ± 0.01	2.5	0.28 ± 0.08	2.9
Thr	0.19 ± 0.04	3.7	0.06 ± 0.01	2.8	0.15 ± 0.01	3.4	0.34 ± 0.08	3.6
Ala	0.69 ± 0.01	13.0	0.29 ± 0.02	14.3	0.68 ± 0.05	15.8	1.89 ± 0.54	19.6
**Tyr**	**0.18 ± 0.01**	**3.3**	**0.05 ± 0.00**	**2.2**	**0.16 ± 0.02**	**3.8**	**0.23 ± 0.03**	**2.4**
**Trp**	**0.06 ± 0.00**	**1.1**	**0.02 ± 0.00**	**0.8**	**0.03 ± 0.01**	**0.7**	**0.04 ± 0.01**	**0.4**
Met	0.02 ± 0.00	0.3	0.01 ± 0.00	0.3	0.02 ± 0.01	0.6	0.03 ± 0.00	0.3
Val	0.11 ± 0.00	2.2	0.05 ± 0.01	2.5	0.07 ± 0.00	1.6	0.14 ± 0.03	1.5
**Phe**	**0.34 ± 0.01**	**6.4**	**0.13 ± 0.01**	**6.1**	**0.22 ± 0.03**	**5.1**	**0.55 ± 0.01**	**5.7**
Ile	0.06 ± 0.00	1.1	0.02 ± 0.00	1.2	0.04 ± 0.00	1.0	0.07 ± 0.01	0.8
Leu	0.04 ± 0.00	0.8	0.03 ± 0.00	1.6	0.07 ± 0.01	1.7	0.15 ± 0.02	1.5
Orn	0.02 ± 0.00	0.4	0.01 ± 0.00	0.7	0.01 ± 0.00	0.3	0.03 ± 0.00	0.3
Lys	0.05 ± 0.00	0.9	0.01 ± 0.00	0.7	0.04 ± 0.01	1.0	0.07 ± 0.02	0.7
Pro	0.49 ± 0.01	9.2	0.22 ± 0.00	10.6	0.20 ± 0.01	4.6	0.47 ± 0.02	4.9
All	5.29 ± 0.08	100.0	2.05 ± 0.21	100.0	4.29 ± 0.25	100.0	9.65 ± 1.83	100.0

Cells were harvested just after inoculation (1 DAI), when resuming active growth (4 DAI), in the exponential phase of growth (7 DAI) and when approaching the stationary phase (10 DAI) (Figure 1A). Amino acid pools were quantified through reverse-phase HPLC, following derivatization with *o*PDA; Pro and total amino acid contents were measured using the ninhydrin method. Cys was undetectable. Mean values ± SEs over six replicates from two independent experiments are reported. Results for AAAs are emphasized in bold.

**Table 2:** Primers used for cDNA amplification and sequencing and for qRT-PCR analysis. Primer pairs were designed using Primer 3 software (http://primer3.ut.ee/ [accessed on 19 July 2019]).

Gene	Primers	cDNA Product Size
cDNA sequencing		
*SHKA*	fwd AGCTGCCGAGTTACAGGGGAGACA rev ATGCACTCTGTGACGTTTTGGCCTG	832
*SHKB*	fwd GAAGCTTGGTGAGGCTGCATTGGG rev GCTCATGCACATCGAAGAAAGCTCG	998
qRT-PCR analysis		
*SHKA*	fwd GCCAACCCCCTTGGGATAAA rev TTGCCCAGCTCTTCTCACTG	180
*SHKB*	fwd TTGACCACCCTATCATGGCG rev CATTTGCATGAGCACCGTCA	177
*Actin 7*	fwd1 TGCTATTCAGGCTGTGCTCT rev1 CGAAGAATGGCATGGGGCAA	129

## Data Availability

The raw data supporting the conclusions of this article will be made available by the authors upon request.

## Supplementary Materials

The following supporting information can be downloaded at: https://www.mdpi.com/article/10.3390/plants12132524/s1, Figure S1: Chromatographic isolation of DAHP synthase from *N. plumbaginifolia* cell extracts. Figure S2: CLUSTAL Omega multiple sequence alignment of the two DAHP synthase isogene families from selected members of *Solanaceae*. Figure S3: Alignment of partial cDNA sequences of the two DAHP synthase isoforms from *Nicotiana plumbaginifolia*. Figure S4: Phylogenetic relationships of wild tobacco DAHP synthase isoforms with the enzymes from other plants.
