# Supplementary material for: The Levels of DAHP Synthase, the First Enzyme of the Shikimate Pathway, Are Related to Free Aromatic Amino Acids and Glutamine Content in Nicotiana plumbaginifolia Cell Cultures
Source: Plants (Basel). 2023 Jul 1;12(13):2524. doi: 10.3390/plants12132524 (PMC10347239; doi:10.3390/plants12132524)
Supplement: Supplementary file 1 [file plants-12-02524-s001.zip › plants-2449145-supplementary materials.pdf]

Article

The levels of DAHP synthase, the first enzyme of the shikimate pathway, are related to free aromatic amino acids and glutamine content in *Nicotiana plumbaginifolia* cell cultures

Giuseppe Forlani [1,*], Samuele Giberti [1] and Enrico Doria [2]

[1] Laboratory of Plant Physiology and Biochemistry, Department of Life Science and Biotechnology, University of Ferrara, Ferrara, Italy; flg@unife.it

[2] Laboratory of Plant Biochemistry, Department of Biology and Biotechnology, University of Pavia, Pavia, Italy; enrico.doria@unipv.it

* Correspondence: flg@unife.it; Tel.: +39 0382 455311, Department of Life Science and Biotechnology, University of Ferrara, via Luigi Borsari 46, I-44121 Ferrara, Italy.

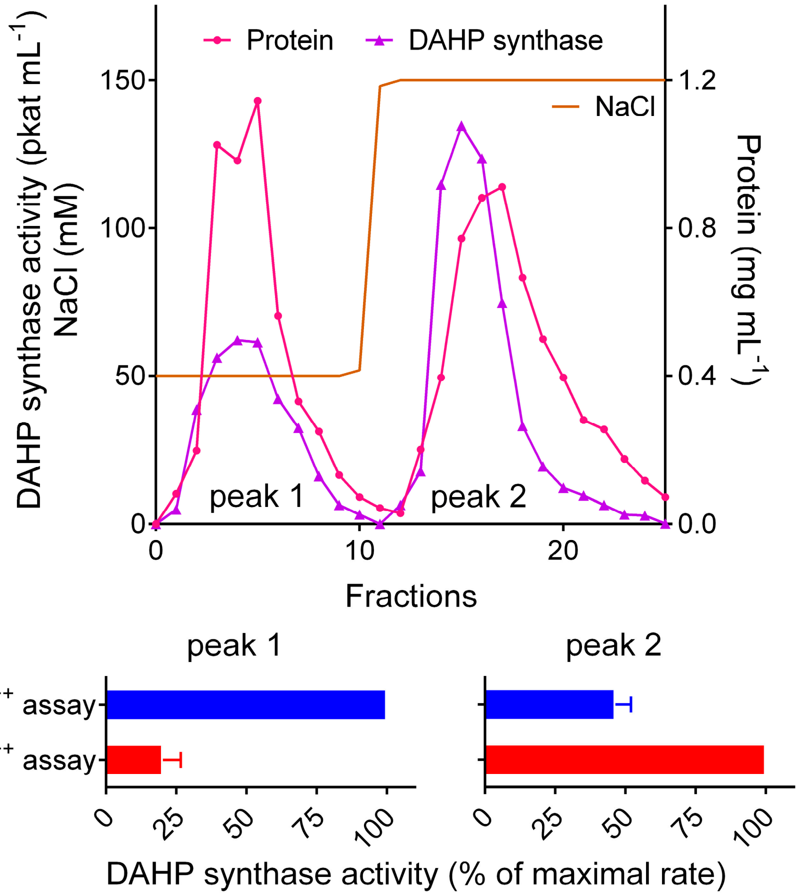

Figure S1. Chromatographic isolation of DAHP synthase from *N. plumbaginifolia* cell extracts. In plant cells a second enzyme exists that is able to catalyse the same reaction *in vitro*, provided that divalent cations such as Co^{++} or Mg^{++} are added to the reaction mixture. DAHP synthase was resolved from the Co^{++}-dependent enzyme, possibly a 3-deoxy-D-manno-octulosonate 8-phosphate synthase, by ion exchange chromatography (upper panel). This was required because both wild tobacco enzymes showed a significant catalytic rate under selective assay conditions (lower panel) that were on the contrary found to discriminate the enzymes from pea. In the case of Co^{++} assay, the reaction mixture contained 100 mM Tris-HCl buffer, pH 8.8, 4 mM glycolaldehyde, 2 mM PEP,

0.5 mM $CoCl_2$, and a limiting amount of enzyme. For Mn^{++} assay, the reaction mixture contained 50 mM EPPS-KOH pH 7.75, 0.5 mM E4P, 2 mM PEP, 0.7 mM $MnCl_2$ and the enzyme. Data in lower panel are means ± SE over 6 replicates obtained with 3 independent enzyme preparations.

```
Nicotiana sylvestris SHKA    ------------------------------------------------------------
        0
Petunia hybrida SHKA         ------------------------------------------------------------
        0
Capsicum annuum SHKA         ----------------------------ccatacacaaaacctcccttat----------
        22
Nicotiana tabacum SHKA       ------------------------------------------------------------
        0
Solanum tuberosum SHKA       ------------------------------------------------------------
        0
Solanum lycopersicum SHKA    ------------------------------------------------------------
        0
Capsicum annuum SHKB         atgggttatgaattcctgaacagtgatggtattatcagaggctctcctatcggtgaggaa
        60
Nicotiana sylvestris SHKB    ------------------------------------------------------------
        0
Nicotiana tabacum SHKB       ------------------------------------------------------------
        0
Solanum tuberosum SHKB       ------------------------------------------------------------
        0
Solanum lycopersicum SHKB    ------------------------------------------------------------
        0

Nicotiana sylvestris SHKA    ------------------------------------------------------------
        0
Petunia hybrida SHKA         ------------------------------------------------------------
        0
Capsicum annuum SHKA         -----------aaaac-------------cccgttccattttccccctcttc--------
        50
Nicotiana tabacum SHKA       ------------------------------------------------------------
        0
Solanum tuberosum SHKA       ------------------------------------------------------------
        0
Solanum lycopersicum SHKA    ------------------------------------------------------------
        0
Capsicum annuum SHKB         gcttgcttggttgggtcctataatgtgatgcaggaaaggtttgggcctattcacaataat
        120
Nicotiana sylvestris SHKB    ------------------------------------------------------------
        0
Nicotiana tabacum SHKB       ------------------------------------------------------------
        0
Solanum tuberosum SHKB       ------------------------------------------------------------
        0
Solanum lycopersicum SHKB    ------------------------------------------------------------
        0

Nicotiana sylvestris SHKA    ------------------------------------------------------------
        0
Petunia hybrida SHKA         -----------------------------------------------------gcagcaa
        7
Capsicum annuum SHKA         -c-tcagg--------taccccatccaccatccatctctctgtctctctctcaaaacaaa
        100
Nicotiana tabacum SHKA       ---------------------------------------ctcaatttcaggtacccaaaa
        21
Solanum tuberosum SHKA       ---------------------------------------------aaaagaggtgtccaa
        15
Solanum lycopersicum SHKA    --------------------------------catctctctccataacacaaggtgccca
        28
Capsicum annuum SHKB         ttttgtgatcagcttgtaggtagtgttgcacagaccagtgggtctgtaattctttatgaa
        180
Nicotiana sylvestris SHKB    ------gatctcctcctccta--------cagatcctctttctctctatttacagaaaaa
        46
Nicotiana tabacum SHKB       --ttcagatctcctcctccca--------tagatcctctttttctctatttacagataca
        50
Solanum tuberosum SHKB       ---------gcgctcctccat--------tgatacctcttcccctttctctctcta----
        39
Solanum lycopersicum SHKB    -------------------at--------tgatacctcttcccctttctctctcta----
        29

Nicotiana sylvestris SHKA    -ctcaggtagtaccacatttgaggcttcacat-cttttccttacaatacacagaaagaat
        58
Petunia hybrida SHKA         caccctccatctcc--tcttgaaaaaaaatcactcacattttgcacaaaagtaccaaaaa
        65
Capsicum annuum SHKA         caaataaaaggtg---tgcccaaaacaaacaaaat-tacacacacattttacattttgtc
        156
Nicotiana tabacum SHKA       aacaagaaaat-----cacataccaaaaaacactc-acatttctcataaaagtattaaat
        75
Solanum tuberosum SHKA       aaagagaaaattacacttcataaactc------tc-acatttgtgttttttcacaaaata
        68
Solanum lycopersicum SHKA    aacaagaaaattacacttcataaatactcacattt-tgtgttgtttttcttcacaaaata
        87
Capsicum annuum SHKB         gtcagtggttttct--ctcctataatatccca----gta------gttcaaagagag-gg
        227
Nicotiana sylvestris SHKB    gtagtca-tcttct--ttttttttatagaga---------------------gaaaaaga
        82
Nicotiana tabacum SHKB       gaaaaagttgtcat--ctttttttataaaga---------------------gaaaaaga
        87
```

```
Solanum tuberosum SHKB          -aaaaagtttgtct--tttttatagtgagaga----aaaaagaggattcaagaatcatca
          92
Solanum lycopersicum SHKB       -aaaaaattgttat--tttatagtgagagaaa----aa---gaggattcaagaatcatca
          79

Nicotiana sylvestris SHKA       ccaatggctctttcaactagcagcg---ccaactccctcctacctaatata---gcctta
          112
Petunia hybrida SHKA            tcaatggctctttcaacaaatagcaccaccagctctcttcttccaaaaacacctttg---
          122
Capsicum annuum SHKA            caaatggctctttcaaatactagcactcccaactcccttttccctaacaaa---tcatta
          213
Nicotiana tabacum SHKA          tcaatggctctttcaagcagtagcactaccaactcccttcttcccaacaaatctcaactg
          135
Solanum tuberosum SHKA          gcaatggctctttcaagtactagcactaccaactctcttctccccaacaga---tctttg
          125
Solanum lycopersicum SHKA       gcaatggctctttcaactaataccactaccaactctcttctttccaacaaa---tctttg
          144
Capsicum annuum SHKB            tgaatc-ccatcaggaccaggggctttatggggcccttgctggttca-gcatgtcccatg
          285
Nicotiana sylvestris SHKB       ggaatcaagaatccagaaaatggctttatcaaataccttatcattg---------tcatc
          133
Nicotiana tabacum SHKB          ggaatcaagaattcagaaaatggctttatcaaacaccttatcattg---------tcatc
          138
Solanum tuberosum SHKB          --aaagctaaaaaataatcatggctttatcaaacaccttatcattg---------tcatc
          141
Solanum lycopersicum SHKB       aaaggcaaaaaaaataatcatggctttatcaaacaccttatcattg---------tcatc
          130
                                  *                  *             *    *

Nicotiana sylvestris SHKA       attcaaactcaacctcttttaccttctcccaaacat------gcccttcccaccaactc-
          165
Petunia hybrida SHKA            gt---------------tcaacaaccccttctaaagaatgcatcttt---accaaccacc
          164
Capsicum annuum SHKA            actcaaaatcaacctctttttccttcccctcttaaaaatg---------------catct
          258
Nicotiana tabacum SHKA          gttcaaaatcaatcccttttaccttctcctctaaagaatgtatctttcaccaccaactca
          195
Solanum tuberosum SHKA          gttcaaaatcaaccccttctgccttctcctttaaagaatgcatttttctccaacaactca
          185
Solanum lycopersicum SHKA       cttcaaaatcaaccccttttgtcttctccttcaaagaatgcatttttctccaacaaatca
          204
Capsicum annuum SHKB            atggaaattgga----------gctgacctggt--cagtggtatagagacccaggaagaa
          333
Nicotiana sylvestris SHKB       atcaaaa-----------------tcccttgtt--caatctcaccttctcca--------
          166
Nicotiana tabacum SHKB          atcaaaa-----------------tcccttgtt--caatctcaccttctcca--------
          171
Solanum tuberosum SHKB          atcaaaa-----------------tcccttgtt--caatctcatcttctcca--------
          174
Solanum lycopersicum SHKB       atcaaaa-----------------tcccttgtt--caatctcatctgctcca--------
          163
                                                           *
Nicotiana sylvestris SHKA       --------aaccaaacccgtt-agaccga--tctcagccgtctattctcccgactcctcc
          214
Petunia hybrida SHKA            accaaagccattagattcatt-caaccaa--tctcagccatccattcatctgattcatca
          221
Capsicum annuum SHKA            ttccccaccgttagattcgtt-caaccaa--tctcagccgttcattcttccgactcttcc
          315
Nicotiana tabacum SHKA          accaaacccgttagatttgtt-caaccaa--tctcagccattcattcctctgactcttcc
          252
Solanum tuberosum SHKA          accaaaaccgttagatttgtt-caaccta--tctcagctgttcattcatctgattctaac
          242
Solanum lycopersicum SHKA       accaaaacggttagatttgtt-caaccta--tcgcagcggttcattcgtctgattcaaac
          261
Capsicum annuum SHKB            ag---aagtgatgtagtcgtggatttcagtgggagcactaaccagaagaatggctttacc
          390
Nicotiana sylvestris SHKB       --------------------c--aacccctcattaccccaaccccgtattcctctttttc
          204
Nicotiana tabacum SHKB          --------------------c--aatccctccttaccccagcctcgtattcccgtttttc
          209
Solanum tuberosum SHKB          --------------------t--aaccc---tttaccccaacctcgtttttccctttttc
          209
Solanum lycopersicum SHKB       --------------------t--aatcc---tacaccccaacctcgtttttccctttttc
          198
                                                          *          *                 *
```

```
Nicotiana sylvestris SHKA     aagaccccctaaaccttcgccg---ccg--gcagccacct------cctccactgctgctg
        263
Petunia hybrida SHKA          aaaaacactcccattgt---------c--tcagccaagccatcatcaccacctgccgcca
        270
Capsicum annuum SHKA          aaaaaccccattgtttccgac---aaa--ccctctaagtcctcctctccggcggcggcca
        370
Nicotiana tabacum SHKA        aagaaccccattgtctccgac---aag--ccctcctccaagccttcaccgccggcggcca
        307
Solanum tuberosum SHKA         aaaatccccattgtttccgat---aag--ccctccaagtcttctccaccggcggccactg
        297
Solanum lycopersicum SHKA     aaaaaccccattgtttccgat---aag--cccaccaagtcttctccaccggcggccactg
        316
Capsicum annuum SHKB          atacaccctttcattgtgatcatcaaaatttcttcaccaccaacatgg------------
        438
Nicotiana sylvestris SHKB     acaacccccaacatgggcggcgccaccccatctccgccgtacatgcggcggagcccgcca
        264
Nicotiana tabacum SHKB        acaacccccaacatgggcggcgccaccccatctccgccgtacacgcggcggagcccgcca
        269
Solanum tuberosum SHKB        cgaccacccaacatgggcggcgccaccccatctccgccgtacatgcggcggagccatcca
        269
Solanum lycopersicum SHKB     cgaccacccaacatgggcggcgccaccctatctccgccgtacacgcggcggagccatcca
        258
                                     *

Nicotiana sylvestris SHKA     ctgtcacggggaaaaaaacagaccaagtttctaaaaaatggtcagttgacagctggagat
        323
Petunia hybrida SHKA          cctccacggcggcggctacggcggtgacaaaacaagaatggtccattgattcttggaaaa
        330
Capsicum annuum SHKA          ccgttacggcggcggctccggcggtgacgaagacggaatgggcggtggatagctggaagt
        430
Nicotiana tabacum SHKA        ctgttacggcggcggctacgacggtgacaaaaacagaatggacagtggagagctggaaat
        367
Solanum tuberosum SHKA        ccaccacggctccggctccggctgtgaccaaaactgaatgggctgtggatagctggaaat
        357
Solanum lycopersicum SHKA     caaccacggctccggctccggctgtgaccaaaactgaatgggcggtggatagctggaagt
        376
Capsicum annuum SHKB          --gcagcgccgccccatctccgccgtgtgctttgccatgcacgcggcggagccagggaag
        496
Nicotiana sylvestris SHKB     aaacagcaactgcttcacagccgttgaaaaaaacccaatggagtcttgattcttggaaaa
        324
Nicotiana tabacum SHKB        aaacagcaactgcttcacagccgttgaaaaaaacccaatggagtcttgattcttggaaaa
        329
Solanum tuberosum SHKB        agactgcagtt---------------aagcaaggaaaatggagtcttgatagctggaaaa
        314
Solanum lycopersicum SHKB     agactgcagtt---------------aagcaaggaaaatggagtcttgatagctggaaaa
        303
                                    *                             *          *      **

Nicotiana sylvestris SHKA     cgaagaaagcacttcagcttccagagtacccgaataaagatgatcttgaatcagttctta
        383
Petunia hybrida SHKA          ccaagaaagctcttcaattacctgaatatccaaatcaagaagagcttaaaaatgttttga
        390
Capsicum annuum SHKA          cgaagaaggctcttcagataccggaatacccgaatcaggaggagcttagatccgttctga
        490
Nicotiana tabacum SHKA        ccaaaaaggctcttcagttacccgaatacccaaatcaagaggagcttcaatctgttctta
        427
Solanum tuberosum SHKA        ctaagaaggcgcttcagctaccggaatacccaaatcaagaggagcttagatctgttctta
        417
Solanum lycopersicum SHKA     ctaagaaggcgcttcagctaccggaatacccagatcaagaggagcttagatctgttctta
        436
Capsicum annuum SHKB          tggagaaggcattgcaacttcctgagtatcgggataagaaagaggtagaatctgtgctgc
        556
Nicotiana sylvestris SHKB     gtaaaaaggctttgcaattacctgaatacccagatgaaaaagaacttgaatcagtgctta
        384
Nicotiana tabacum SHKB        gcaaaaaggctttgcaattacctgaatacccagatgaaaaagaacttgaatctgtgcttg
        389
Solanum tuberosum SHKB        ccaagaaagctttgcaacttcctgaatacccagatgagaaagaacttgaatctgtgctta
        374
Solanum lycopersicum SHKB     caaagaaagctttacaacttcctgaatacccagatgagaaagaacttgaatcggtgctta
        363
                                 * ** **  * **  * ** ** ** *   ** *  * **  *   *   **  *

Nicotiana sylvestris SHKA     agaccattgaagatttccctccaattgtgtttgctggtgaggcgaggagtcttgaagagc
        443
Petunia hybrida SHKA          aaacaattgaagatttcccaccaattgtatttgctggtgaagcaaggcatcttgaagaaa
        450
Capsicum annuum SHKA          agacgattgaggagtttcctcctattgtttttgctggtgaagcgaggagccttgaagagc
        550
Nicotiana tabacum SHKA        agacgattgaagagttccctcctatcgtgtttgctggtgaggcgagaagtcttgaggagc
        487
Solanum tuberosum SHKA        agacgatcgatgagttccctcctatcgtgtttgctggtgaggctaggagccttgaagaac
        477
Solanum lycopersicum SHKA     agacgatcgatgagttccctcctatcgtctttgctggtgaggctagaagccttgaagaac
        496
Capsicum annuum SHKB          aaacatttgaaatgaatccaccatttgtgtttgctggtgatgcaaggagtttggaaaaga
        616
Nicotiana sylvestris SHKB     aaactcttgaatctaaccctccacttgtgtttgctggtgaagctaggaatttagaagaga
        444
Nicotiana tabacum SHKB        aaactcttgaatctaatcctccacttgtgtttgctggtgaagctaggaatttagaagaga
        449
Solanum tuberosum SHKB        aaacacttgaaatgaatccaccacttgtgtttgctggtgaggcaaggagtttggaagaga
        434
Solanum lycopersicum SHKB     aaacacttgaaatgaatccaccccttgtgtttgctggtgaggcaaggagtttggaagaga
        423
                              * **  * **       ** **  * ** *********** ** **     * **  *
```

```
Nicotiana sylvestris SHKA   gacttggagaggctgcaatggggaaggcgtttctgctacaaggtggtgactgtgccgaga
          503
Petunia hybrida SHKA        agcttggtgaagctgctatgggaagagcctttttgttacaaggtggtgattgtgctgaga
          510
Capsicum annuum SHKA        ggcttgctgaggcggctatggggagggcgtttttgttgcaaggaggtgattgtgctgaga
          610
Nicotiana tabacum SHKA      gtctcggtgaggctgctatgggccgggctttcttgttacaaggaggtgattgtgctgaga
          547
Solanum tuberosum SHKA      gccttggtgaggctgctatgggaagggctttttttgttacaaggaggagattgtgctgaga
          537
Solanum lycopersicum SHKA   gacttggtgaggctgctatgggaagggcgtttttgttacaaggaggagattgtgccgaga
          556
Capsicum annuum SHKB        agattggtgaagctgcattgggtaaagctttttttgttgcaaggtggtgattgtgctgaga
          676
Nicotiana sylvestris SHKB   aacttggtgaagctgctttaggaaaagctttttttattacaaggtggtgattgtgctgaga
          504
Nicotiana tabacum SHKB      agcttggtgaagctgctttaggaaaagctttttttattacaaggtggtgattgtgctgaga
          509
Solanum tuberosum SHKB      agcttggtgaggctgcattggggaaagctttttttgttacaaggtggagattgtgctgaga
          494
Solanum lycopersicum SHKB   agcttggtgaggctgcattggggaaagctttttttgttacaaggggagactgtgctgaga
          483
                               * *  ** ** **  * **    ** **  *  * ***** ** ** ***** ****
Nicotiana sylvestris SHKA   gtttcaaggaattcaatgctgttaacataagggacactttcagaatcatccttcaaatgg
          563
Petunia hybrida SHKA        gttttaaggaatttaatgctaataacataagggatacttttagaatccttcttcaaatgg
          570
Capsicum annuum SHKA        gctttaaggaattcaacgcgaataacattagggatacgtttagaattcttcttcaaatgg
          670
Nicotiana tabacum SHKA      gttttaaggaatttaatgccaataatattagggatacttttagaatccttcttcaaatgg
          607
Solanum tuberosum SHKA      gtttcaaggaattcaatgctaataatattagggatactttcagaatcctccttcaaatgg
          597
Solanum lycopersicum SHKA   gtttcaaggaattcaatgctaataatattagggatactttcagaatccttcttcaaatgg
          616
Capsicum annuum SHKB        gttttaaggagtttaatgcgaataattttcgtgatacttttaggattatacttcagatga
          736
Nicotiana sylvestris SHKB   gttttaaggaatttaatgctaataatattcgtgatacttttaggattcttcttcagatga
          564
Nicotiana tabacum SHKB      gttttaaggaatttaatgctaataatattcgtgatacttttaggattcttcttcagatga
          569
Solanum tuberosum SHKB      gttttaaggaatttaatgccaataatattcgtgatacttttaggattttgcttcagatga
          554
Solanum lycopersicum SHKB   gttttaaggagtttaatgccaataatattcgtgatactttcaggattttgcttcagatga
          543
                            * ** ***** ** ** **   ***  *  * ** ** ** ** **  * ***** ***

Nicotiana sylvestris SHKA   gtgctgttcttatgttcggtggtcagatgcccgttgtcaaggttggaagaatggctgggc
          623
Petunia hybrida SHKA        gtgctgttcttatgtttggtggtcagatgcctgttatcaaggtgggaagaatggctggac
          630
Capsicum annuum SHKA        gtgctgttcttatgtttggtggtcaagtacctgtgatcaaggttggaagaatggctgggc
          730
Nicotiana tabacum SHKA      gtgctgttctcatgtttggtggtcagatgcctgttatcaaggttggaaggatggctgggc
          667
Solanum tuberosum SHKA      gtgctgttcttatgtttggcggtcagatgcctgtgatcaaggttggaagaatggctgggc
          657
Solanum lycopersicum SHKA   gtgctgttcttatgtttggtggtcagatgcctgtgatcaaggttggaagaatggctgggc
          676
Capsicum annuum SHKB        gtgttgttcttatgtttggtggtgaagttcctgtcactaaggttggaagaatggcgggtc
          796
Nicotiana sylvestris SHKB   gtgttgttcttatgtttggtggtcaagttcctgttattaaggttggaagaatggcgggtc
          624
Nicotiana tabacum SHKB      gtgttgttcttatgtttggtggtcaagttcctgtgattaaggttggaagaatggcgggtc
          629
Solanum tuberosum SHKB      gtgttgttcttatgtttggtggacaagttcctgtgattaaggttggaagaatggcgggtc
          614
Solanum lycopersicum SHKB   gtgttgttcttatgtttggtggacaagttcctgtcattaaggttggaagaatggccggtc
          603
                            *** ****** ***** ** **  *  * ** **    ***** ***** ***** ** *
```

```
Nicotiana sylvestris SHKA 683   aatttgcaaagccaagatcagactcatttgaagagaaagatggagtaaagctgccaagtt
Petunia hybrida SHKA 690        aatttgcaaagccaagatcagataactttgaggagaagaatggtgttaagttgcctagtt
Capsicum annuum SHKA 790        aatttgcaaagccaagatcagatccatttgaggagaaggatggtgtaaagctgccgagtt
Nicotiana tabacum SHKA 727      aatttgcaaagccaagatcagataattttgaggagaagaatggagtgaagctgccgagtt
Solanum tuberosum SHKA 717      aatttgcaaagccaagatcagattcatttgaggagaaggatggtgtaaagctgccgagtt
Solanum lycopersicum SHKA 736   aatttgcaaagccgagatcagattcatttgaggagaaggatggtgtaaagctgccgagtt
Capsicum annuum SHKB 856        agtttgcaaaaccaagatcagatccgtttgaggagataaacggagtgaagttaccaagtt
Nicotiana sylvestris SHKB 684   agtttgcaaaaccaagatcagatccatttgaggagattgatggagtgaagctgccaagtt
Nicotiana tabacum SHKB 689      agtttgcgaaaccaagatcagatccgtttgaggagattgatggagtgaagctgccaagtt
Solanum tuberosum SHKB 674      agtttgcaaaaccaagatcagatccgttggaggagataaatggagtgaagctaccaagtt
Solanum lycopersicum SHKB 663    agtttgcaaaaccaagatcagatccgtttgaggagataaatggagtgaagctaccaagtt
                                * ***** ** ** ********    ** ** ****   * ** ** *** * ** ****

Nicotiana sylvestris SHKA 743   acaggggagataatgtgaatggagatgcatttgatgaaaaatccagaattccggatcctc
Petunia hybrida SHKA 750        acaggggagacaatgtgaatggagacgcatttgatttgaagtccaggactcctgatcctc
Capsicum annuum SHKA 850        acaggggagacaatgtgaatggagatgcatttgattccaagtccaggactcctgatcctc
Nicotiana tabacum SHKA 787      acaggggagacaacgtgaacggagatgcatttgatgccaagtcaagaactcctgaccctc
Solanum tuberosum SHKA 777      acaggggagacaatgtcaacggagatgcatttgatgtcaagtccaggactcctgaccccc
Solanum lycopersicum SHKA 796   acaggggagacaatgtgaatggagatgcatttgatgtcaagtccaggactcctgaccctc
Capsicum annuum SHKB 916        acaagggcgacaatatcaatggtgatacatttgatgagaagtcgagaaccccagacccat
Nicotiana sylvestris SHKB 744   acaagggtgataatattaatggtgatacatttgatgagaagtcaagaattccagaccctc
Nicotiana tabacum SHKB 749      acaagggtgataacattaatggcgatacatttgatgagaagtcaagaattccagaccctc
Solanum tuberosum SHKB 734      acaagggtgacaatatcaatggtgatacatttgatgagaagtcaagaattccggatcctc
Solanum lycopersicum SHKB 723   acaagggtgacaatatcaatggtgatacgttcgatgagaagtcaagaattccagatcctc
                                *** *** ** **  * ** ** **  * ** ***   ** ** ** *  ** ** **

Nicotiana sylvestris SHKA 803   aaaggatgatcagggcctattgtcaatctgcagctactttgaatctcttgagggcctttg
Petunia hybrida SHKA 810        agaggctgatcagggcttattgccaatctgcagctactttgaatctattgagggcttttg
Capsicum annuum SHKA 910        agagactgatcagggcctactgccaatctgctgctactttgaatctattgagggctttcg
Nicotiana tabacum SHKA 847      agaggttgatcagggcgtattgtcaatctgcagctactttgaatctattgagggcttttg
Solanum tuberosum SHKA 837      agaggctgatcagggcctattgccaatctgcagctactttgaatctgttgagggctttcg
Solanum lycopersicum SHKA 856   agaggctgatcagggcctattgccaatctgcagctactttgaatctgttgagggcttttg
Capsicum annuum SHKB 976        ataggcttattagggcctacatgcaatctgccgcgactcttaaccttcttagagctattg
Nicotiana sylvestris SHKB 804   ataggcttattagggcttacatgcaatctgctgcgactcttaaccttcttagggcttttg
Nicotiana tabacum SHKB 809      ataggcttattagggcttacatgcaatctgctgcgactcttaaccttcttagggcttttg
Solanum tuberosum SHKB 794      ataggcttattagggcatacatgcaatctgctgcgactcttaatcttcttagagcttttg
Solanum lycopersicum SHKB 783   ataggcttattagggcatacatgcaatctgctgcgactcttaatcttcttagagcttttg
                                * **  * ** ***** **    ******** ** *** * ** **  * ** **  * *

Nicotiana sylvestris SHKA 863   ctaagggaggatatgctgccatgcagaggatcaaccaatggaacttagattttacagagc
Petunia hybrida SHKA 870        ctactggaggatatgctgccatgcagagggtcactcaatggaacttggatttcacagagc
Capsicum annuum SHKA 970        ctacgggaggatatgctgccatgcagagagtcacacagtggaacttggatttcactgagc
Nicotiana tabacum SHKA 907      ctacaggaggatatgctgccatgcagaggatcaaccaatggaacttggatttcacagagc
Solanum tuberosum SHKA 897      ctactggaggatatgctgccatgcagaggatcaatcagtggaacttggatttcacagagc
Solanum lycopersicum SHKA 916   ctactggaggatatgctgccatgcagaggatcaatcagtggaacttggatttcacagagc
Capsicum annuum SHKB 1036       ctactggaggttatgctgcaatgcagagggtccgtgaatgtaatcttgattttgtggaga
Nicotiana sylvestris SHKB 864   ctactggaggttatgctgcaatgcagagggtcaccgaatggaatcttgattttgtggaga
Nicotiana tabacum SHKB 869      ctactggaggttatgctgcaatgcagagggtcaccgaatggaatcttgattttgtggaga
Solanum tuberosum SHKB 854      ctactggaggttatgctgcaatgcagcgggtcaccgaatggaatcttgattttgtggaga
Solanum lycopersicum SHKB 843   ctactggaggttatgctgcaatgcagagggtcactgaatggaatcttgattttgtggaga
                                ***  ***** ******** ****** *  **    * ** **  * *****    ***
```

```
Nicotiana sylvestris SHKA     acagcgagcagggtgatcggtatcgtgaattagctcatagagtggatgaggcccttggtt
        923
Petunia hybrida SHKA          atagtgagcagggtgataggtaccgagaactagctaacagagtggatgaggcccttgggt
        930
Capsicum annuum SHKA          atagtgagcagggtgataggtatcgtgaactagctaatagagtggatgaggcacttggtt
        1030
Nicotiana tabacum SHKA        acagtgagcagggtgataggtatcgtgaactagctaatagagtggatgaggcccttggtt
        967
Solanum tuberosum SHKA        atagtgagcagggtgataggtaccgtgaactagctagtagagtggatgaggcccttggtt
        957
Solanum lycopersicum SHKA     atagtgagcagggtgataggtaccgtgaactagctagtagagtggatgaggcccttggtt
        976
Capsicum annuum SHKB          acagtgagcagggcgataggtatcaagagctagctcacagggttgatgaggccttaggat
        1096
Nicotiana sylvestris SHKB     acagtgagcaaggagataggtatcaagaactagctcacagggttgatgaagccttgggat
        924
Nicotiana tabacum SHKB        acagtgagcaaggagataggtatcaagaactagctcacagagtcgatgaagccttgggat
        929
Solanum tuberosum SHKB        actgtgagcagggagataggtatcaggaactagctcacagggttgatgaagccttaggat
        914
Solanum lycopersicum SHKB     acagtgaacagggagataggtatcaggaactagctcacagggttgatgaagccttaggat
        903
                              *  * ** ** ** *** **** *  **  *****   ** ** ***** **  * ** *

Nicotiana sylvestris SHKA     tcatggctgctgctggacttacaacggaccatcctatcatgaaaaccacggagttttgga
        983
Petunia hybrida SHKA          tcatgaatgctgctggacttacaacggaccatcctatcatgacaaccaccgagttctgga
        990
Capsicum annuum SHKA          tcatggccgcagctggacttacaatggaacatcctattatgaaaaccaccgagttctgga
        1090
Nicotiana tabacum SHKA        tcatggctgctgctggacttacagtggatcatcctattatgaaaaccacagagttctgga
        1027
Solanum tuberosum SHKA        tcatgactgcagctggacttacaatggaccatcctattatgaaaaccactgagttctgga
        1017
Solanum lycopersicum SHKA     tcatgactgcagctggacttacaatggaccatcctattatgaaaaccactgagttctgga
        1036
Capsicum annuum SHKB          tcatgactgctgctggactcacagtcgaccacccaatcatgtcaatgattggattctgga
        1156
Nicotiana sylvestris SHKB     tcatggctgctgctggactcacagttgaccaccctatcatggcgacaactgatttctgga
        984
Nicotiana tabacum SHKB        tcatggctgctgctggactcacagtagaccaccctatcatggcaacaactgatttttgga
        989
Solanum tuberosum SHKB        tcatggctgctgctggactcacagttgaccaccctatcatgtcaacaactgatttctgga
        974
Solanum lycopersicum SHKB     tcatggctgctgctggactcacagttgaccaccctatcatgtcaacaactgatttctgga
        963
                              *****   ** ******** ***   ** ** ** ** ***   *  *  *  ** ****

Nicotiana sylvestris SHKA     cgtctcacgagtgcttacttttgccgtatgagcagtcacttacaagattggattcaactt
        1043
Petunia hybrida SHKA          catctcatgagtgcttacttttgccctatgagcagtcactaactcgattggattcgactt
        1050
Capsicum annuum SHKA          catctcatgagtgcttacttttgccctatgagcagtcactaacacggttggattcaactt
        1150
Nicotiana tabacum SHKA        cttctcatgagtgcttacttttgccctatgagcagtcactaacacgattggattcaactt
        1087
Solanum tuberosum SHKA        catctcatgagtgcttacttttgccctatgagcagtcactaacacgtcgggattcaactt
        1077
Solanum lycopersicum SHKA     catctcatgagtgcttacttttgccctatgaacagtcactaacacgccgggattcaactt
        1096
Capsicum annuum SHKB          catcccacgagtgcttgcttcttccttatgaacaagcacttacacgagaggattcaactt
        1216
Nicotiana sylvestris SHKB     catcccatgagtgcttgcttcttccttatgaacaagcacttacaagggaggattcaactt
        1044
Nicotiana tabacum SHKB        catctcacgagtgcttgcttcttccttatgaacaagcacttacaagggaggattcaactt
        1049
Solanum tuberosum SHKB        catcccacgagtgcttgcttcttccttatgaacaagcacttacaagggaggattcaactt
        1034
Solanum lycopersicum SHKB     catcccatgagtgcttgcttcttccttatgaacaagcacttacaagagaggattcaactt
        1023
                              * ** ** ******** *** * ** ***** **  **** **  *   ****** ****
```

```
Nicotiana sylvestris SHKA   ctggcctttactatgattgctctgcccattttctttgggctggggagagaaccagacaat
             1103
Petunia hybrida SHKA        ctggcctttactatgattgctctgcccattttctttgggttggagagagaactaggcagt
             1110
Capsicum annuum SHKA        ccggcctttactatgattgctctgcccatatgctttgggttggagagagaaccaggcagt
             1210
Nicotiana tabacum SHKA      ctggcctttactatgattgttccgcccattttatttgggttggagaaagaactaggcagt
             1147
Solanum tuberosum SHKA      ctggcctttactatgattgctctgctcatttcctttgggttggagagagaaccaggcagt
             1137
Solanum lycopersicum SHKA   ctggccttcactatgattgctctgctcatttcctttgggttggagagagaaccaggcagt
             1156
Capsicum annuum SHKB        ctggtcttttctatgattgttcggctcacatgatttgggttggggaacgaaccaggcaac
             1276
Nicotiana sylvestris SHKB   ctggtcttttctatgattgttccgctcacatgatttgggttggtgaacgaaccaggcaac
             1104
Nicotiana tabacum SHKB      ctggtcttttctatgattgttccgctcacatgatttgggttggggaacgaaccaggcaac
             1109
Solanum tuberosum SHKB      ctggtcttttctatgattgttctgctcacatggtttgggttggggagcgaaccaggcaac
             1094
Solanum lycopersicum SHKB   ctggtcttttctacgattgttctgctcacatggtttgggttggggaaagaaccaggcaac
             1083
                            * ** ***  *** ***** ** ** **  *  ****** *** **  **** ** **

Nicotiana sylvestris SHKA   tggatggtgcccatgttgagttcttgagaggaattgccaaccccttggtataaaggtga
             1163
Petunia hybrida SHKA        tggatggtgcccatgtcgagttcttgagaggaatcgccaaccccttggtattaaggtga
             1170
Capsicum annuum SHKA        tggatggtgcccatgttgagttcttgagaggagttgccaaccctctcggtattaaggtga
             1270
Nicotiana tabacum SHKA      tggatggtgcccatgttgagttcttgagaggagttgccaaccccttggtattaaggtga
             1207
Solanum tuberosum SHKA      tggatggtgcccatgttgagttcttgagaggaattgccaaccctctcggtattaaggtga
             1197
Solanum lycopersicum SHKA   tggatggtgcccatgttgagttcttgagaggaattgccaaccctctcggtattaaggtga
             1216
Capsicum annuum SHKB        tagatggtgctcatgtcgagtttttgagaggagcagcaaatccgcttggcataaaggtga
             1336
Nicotiana sylvestris SHKB   ttgacggtgctcatgttgagttcttgagaggagtagcaaacccacttggcatcaaggtga
             1164
Nicotiana tabacum SHKB      ttgacggtgctcatgttgagttcttgagaggagtagcaaacccacttggcataaaggtga
             1169
Solanum tuberosum SHKB      tagacggtgctcatgtcgagtttttgagaggagttgcaaaccctcttggcataaaggtga
             1154
Solanum lycopersicum SHKB   tagacggtgctcatgtcgagtttttgagaggagttgcgaaccctcttggaataaaggtga
             1143
                            * ** ***** ***** ***** *********   ** ** ** ** ** ** *******

Nicotiana sylvestris SHKA   gtgacaagatggatccaaatgagttagttaagctcattgacattctgaaccctaacaaca
             1223
Petunia hybrida SHKA        gtgacaagatggacccaagtgcattggtcaagctcattgagattttgaaccctcaaaaca
             1230
Capsicum annuum SHKA        gtgacaagatggatccaaatgcattggtcaagctcatcgagattttgaaccctcaaaaca
             1330
Nicotiana tabacum SHKA      gtgacaagatggatccaagtgcattggtcaagctcattgagattttgaaccctgataaca
             1267
Solanum tuberosum SHKA      gtgacaagatggacccaagtgcattggtcaagctcattgagattttgaacccacaaaaca
             1257
Solanum lycopersicum SHKA   gtgacaagatggacccaagtgcattggtcaagctcattgagattttgaacccacaaaaca
             1276
Capsicum annuum SHKB        gccaaaagatggatccaagtgagctaattaagctcattgacatcctgaacccaaccaata
             1396
Nicotiana sylvestris SHKB   gccaaaagatggatccaaatgagctagttaaactcattgacatcctgaacccaaccaata
             1224
Nicotiana tabacum SHKB      gccaaaagatggatccaaatgagctcgttaaactcattgacatcctgaacccaaccaata
             1229
Solanum tuberosum SHKB      gtcaaaaaatggatccaaatgagctaattaagctcatagacatcctgaacccagccaata
             1214
Solanum lycopersicum SHKB   gtcaaaaaatggatccaaaagagctaattaagctcattgacatcctgaaccctgccaata
             1203
                            *  * ** ***** ****  *   *  * ** ***** ** **  *******    ** *
Nicotiana sylvestris SHKA   aaccaggaaggattacaataattaccagaatgggagcagagaacatgagggttaagcttc
             1283
Petunia hybrida SHKA        aggctggtagaattacaataattaccagaatgggagcagaaaacatgagggttaagcttc
             1290
Capsicum annuum SHKA        aagctgggaggattacaataattaccagaatgggagcagaaaacatgagggttaagcttc
             1390
Nicotiana tabacum SHKA      aagctgggaggattacaataattaccagaatgggagcagagaacatgagggttaagcttc
             1327
Solanum tuberosum SHKA      aagctggaaggattacaataattaccagaatgggagcagaaaacatgagggttaaacttc
             1317
Solanum lycopersicum SHKA   aagctggaaggattacaataattaccagaatgggagcagaaaacatgagggttaagcttc
             1336
Capsicum annuum SHKB        agcctggaagaataactgtaatcgcgagaatgggtgctgagaacatgagagagaaacttc
             1456
Nicotiana sylvestris SHKB   agcccggaagaattactgtaattgtgagaatgggtgctgagaatatgagagtgaagcttt
             1284
Nicotiana tabacum SHKB      agcccggaagaattactgtaattgtgagaatgggtgctgagaatatgagagtgaagcttt
             1289
Solanum tuberosum SHKB      agcctggaagaattactgtaattgttagaatgggtgctgagaacatgagagtgaaacttt
             1274
Solanum lycopersicum SHKB   agcctggaagaattactgtaattgttagaatgggtgccgagaacatgagagtaaaacttt
             1263
                            *  * ** ** ** **  ****    ******** ** ** ** ***** *  ** ***
```

```
Nicotiana sylvestris SHKA      ctcatctaataagggcagtgagaagagctgggcaaattgttacatgggtatctgatccta
                 1343
Petunia hybrida SHKA           ctcatcttatcagggctgtccgaggagcagggcaaattgtcacttgggtatctgatccta
                 1350
Capsicum annuum SHKA           ctcatcttatcagggcagtccgaagagcagggcaaatcgtcacttgggtatctgatccta
                 1450
Nicotiana tabacum SHKA         ctcatcttatcagagcagtccgaagagcagggcaaatcgtcacttgggtatctgatccta
                 1387
Solanum tuberosum SHKA         ctcatcttatcagggcagtccgaagagcaggacaaattgtgacttgggtatctgatccta
                 1377
Solanum lycopersicum SHKA      ctcatcttatcagggcagtccgaagagcaggacaaattgtgacttgggtatctgatccta
                 1396
Capsicum annuum SHKB           cgcacttggtcagggcagtacgaggagctggtcagattgtggcgtgggtctgtgacccaa
                 1516
Nicotiana sylvestris SHKB      gccacttgatcagggctgttcgaggagctggacagattgttacctgggtttgtgacccaa
                 1344
Nicotiana tabacum SHKB         gccacttgatcagggcagttcgaggagctggacagattgttacctgggtttgtgacccga
                 1349
Solanum tuberosum SHKB         cccacttggtcagggcagtacgaggagctggtcagattgtgacatgggtttgtgatccaa
                 1334
Solanum lycopersicum SHKB      cccacttggtcagggctgtacggggcgctggtcagattgtgacatgggtttgtgatccaa
                 1323
                                 **  *  * ** ** **  *  * ** ** ** ** **  * ***** * *** ** *

Nicotiana sylvestris SHKA      tgcatggaaataccattaaagcaccttgtggtctgaaaactcgaccttttgattccatca
                 1403
Petunia hybrida SHKA           tgcatggaaataccatcaaagctccgtgtggtcttaaaactcgacctttcgattccatca
                 1410
Capsicum annuum SHKA           tgcacggaaataccatcaaagctccttgcggtctaaaaactcgacctttcgattccatca
                 1510
Nicotiana tabacum SHKA         tgcatgggaataccatcaaagctccttgcggtctaaaaactcgaccttttgattccatca
                 1447
Solanum tuberosum SHKA         tgcacggaaataccatcaaggctccttgtggtctaaaaactcgaccttttgattccatca
                 1437
Solanum lycopersicum SHKA      tgcacggaaataccatcaaagctccatgcggtctaaaaactcgaccttttgattccatca
                 1456
Capsicum annuum SHKB           tgcacgggaacaccatcaaggcaccatctggactcaaaactcgtgctttcgatacaatcc
                 1576
Nicotiana sylvestris SHKB      tgcacggaaacactataaaggcaccatgcggactcaaaacccgtgctttcgattcaatcc
                 1404
Nicotiana tabacum SHKB         tgcacggcaacaccataaaggcaccatgcggactcaaaacccgtgctttcgattcaatcc
                 1409
Solanum tuberosum SHKB         tgcacgggaataccatcaaggcaccatgtggactcaaaacccgtgctttcgattcaatcc
                 1394
Solanum lycopersicum SHKB      tgcacgggaataccatcaaggcaccatgtggactcaaaacccgtgctttcgattcaatcc
                 1383
                               **** ** ** ** ** ** ** ** *  ** ** ***** **  **** *** * ***

Nicotiana sylvestris SHKA      gggctgaagtgagagcattcttgacgtgcatgagcaagaaggaagccatccaggaggag
                 1463
Petunia hybrida SHKA           gggctgaagtaagagcattcttgatgttcatgaacaagaaggaagccacccaggaggag
                 1470
Capsicum annuum SHKA           gggctgaagtaagagcattcttgatgttcatgagcaagaaggcagccacccaggaggag
                 1570
Nicotiana tabacum SHKA         gggctgaagtaagagcattcttgatgttcatgagcaagaaggaagccacccaggaggag
                 1507
Solanum tuberosum SHKA         gggccgaggtaagagcattcttgatgttcatgatcaagaaggaagccacccaggaggag
                 1497
Solanum lycopersicum SHKA      gggctgaggtaagagcattcttgatgttcatgatcaagaaggaagccacccaggaggag
                 1516
Capsicum annuum SHKB           tggctgaagttcgagccttcttcgatgtgcatgagcaagaagggagccatcctggtggca
                 1636
Nicotiana sylvestris SHKB      tggctgaggttcgagctttcttcgatgtgcatgagcaagaagggagccaccctggtggta
                 1464
Nicotiana tabacum SHKB         tggctgaggtccgagctttcttcgatgtgcatgagcaagaagggagccaccctggtggta
                 1469
Solanum tuberosum SHKB         tggctgaggtccgagctttcttcgatgtgcatgagcaagaaggaagccaccctggtggta
                 1454
Solanum lycopersicum SHKB      tggctgaggtccgagctttcttcgatgtgcatgagcaagaaggaagccaccctggtggta
                 1443
                                *** ** **  **** ***** ** ** ***** ******** ***** ** ** **
```

```
Nicotiana sylvestris SHKA 1523     ttcacctagagatgacaggccaaaatgtaacagagtgcattggtggatcaaggaccgtga
Petunia hybrida SHKA 1530          tgcaccttgagatgactggtcaaaacgtcacagagtgcatcggtggatcaagaactgtga
Capsicum annuum SHKA 1630          tccaccttgagatgacaggccaaaatgtcacagagtgcatcggtggatcaagaaccgtga
Nicotiana tabacum SHKA 1567        tacacctggagatgacaggccaaaacgtcacagagtgcattggtggatcacgaactgtga
Solanum tuberosum SHKA 1557        ttcatctggagatgacaggccaaaacgtcacagagtgcatcggtggatcacgaactgtga
Solanum lycopersicum SHKA 1576     ttcatcttgagatgacaggccaaaacgtcacagagtgcattggtggatcacgaactgtga
Capsicum annuum SHKB 1696          tacatctagaaatgacagggcaaaatgtgactgaatgcaatggtggatcacgaatagtga
Nicotiana sylvestris SHKB 1524     tccatctagaaatgacagggcaaaatgtgactgaatgcattggcggatcacgaacagtaa
Nicotiana tabacum SHKB 1529        tccatctagaaatgacagggcaaaatgtgactgaatgcattggcggatcacgaacagtaa
Solanum tuberosum SHKB 1514        ttcacctagaaatgacagggcaaaatgtgactgagtgcattggtggatcacgaacagtaa
Solanum lycopersicum SHKB 1503     ttcaccttgaaatgacagggcaaaatgtgactgaatgcattggtggatcacgaacagtaa
                                   * ** ** ** ***** ** ***** ** ** ** ****  ** ****** * *  ** *

Nicotiana sylvestris SHKA 1583     ctttcgatgatctgagctcacgctatcacacccactgcgatcctagactcaatgcatctc
Petunia hybrida SHKA 1590          ccttcgatgatctgagctcacgttaccacacccactgtgatcctaggctcaacgcatctc
Capsicum annuum SHKA 1690          cctttgatgatctgagctcacgttaccacacccactgtgatcctaggctcaatgcatctc
Nicotiana tabacum SHKA 1627        cctttgatgatctgagctcacgttaccacacccactgtgatcctaggctcaatgcatctc
Solanum tuberosum SHKA 1617        cctttgatgatctgagctcacgttaccacactcactgtgatcctaggctcaatgcatctc
Solanum lycopersicum SHKA 1636     cctttgatgatctgagctcacgttaccacactcactgtgatcctaggctcaatgcatctc
Capsicum annuum SHKB 1756          cctatgacgatttgagctctcgctaccacacacattgtgacccgagattgaatgcttctc
Nicotiana sylvestris SHKB 1584     cctacgacgatttgggctctcgctaccacacacattgtgacccaagattgaatgcttccc
Nicotiana tabacum SHKB 1589        cctacgacgatttgggctctcgctaccacacacattgtgacccaagattgaatgcttccc
Solanum tuberosum SHKB 1574        cctatgacgatttgggctctcgctaccacactcattgtgacccaagattgaatgcttctc
Solanum lycopersicum SHKB 1563     cctatgacgatttgggctctcgctaccacactcattgtgacccaagattgaatgcatctc
                                   * *  ** *** ** **** ** ** ***** ** ** ** ** **  * ** ** ** *
Nicotiana sylvestris SHKA 1643     aatcccttgaactcgcctttattatcgctgaacgcctcagaaagaggaggcttggatctc
Petunia hybrida SHKA 1650          aatcccttgaactcgcgttcattattgcagaacgccttagaaaaggagacttggatcac
Capsicum annuum SHKA 1750          aatcccttgagctctcattcattatcgcagaacgtttgagaaaaggaggcttggatcac
Nicotiana tabacum SHKA 1687        aatcccttgagctcgccttcattattgcagaacgcctaagaaagaggaggcttggatcac
Solanum tuberosum SHKA 1677        aatcccttgagctctcgttcattatcgcagaacgtttgaggaaaggaggctcggatcca
Solanum lycopersicum SHKA 1696     aatcccttgagctctcattcattatcgcagaacgtttgaggaaaggaggctcggatcgc
Capsicum annuum SHKB 1816          agtcgcttgaactttccttcatcatagctgaacaactaagaaggcgaagaacagccactc
Nicotiana sylvestris SHKB 1644     aatctcttgaactttccttcatcgtagctgaacgactaagaaaacgaagaatggccactc
Nicotiana tabacum SHKB 1649        aatctcttgaactttccttcatcgtagctgaacgactaagaaaacgaagaatggcctctc
Solanum tuberosum SHKB 1634        agtctcttgaactttccttcatcgtagctgagagactaagaagacgaagaatgtccactc
Solanum lycopersicum SHKB 1623     agtctcttgaactctccttcatcgtagctgagcgactaagaagaagaagaatgtcatctc
                                   * ** ***** **  * ** **  * ** **     * ** *   * **        *

Nicotiana sylvestris SHKA 1703     aaagctcattaagttactaaatatgtagaaagcttacgttgctatgttgacaaaatttct
Petunia hybrida SHKA 1709          aaagcgtgttaggtcaatagatacgtggaaaggttcaaatgctgttttcttggtt-ttac
Capsicum annuum SHKA 1810          aaagcgtattaggtcaatagatatttggaaatgttcaaatgttgctgccttttatattcc
Nicotiana tabacum SHKA 1746        aaaacgtattaggtcaatagatatttggaaaggtccaaaatgctgcttttcttga-ttta
Solanum tuberosum SHKA 1735        -aagcgttttagggcaatagatatttggaaatgttcaagtactgcctttttctat-ttac
Solanum lycopersicum SHKA 1755     aaagcacattagggcaatagatatttggaaatgttcaagtactgccttttttat-ttac
Capsicum annuum SHKB 1871          aacgcgtgtaggcgtttagtctgttgtcttgaaacttttgcttttggcatttt-----ga
Nicotiana sylvestris SHKB 1703     aacgcctgtaggctttctctaatttacccg-tatttttagtctgcatccttgttctcttg
Nicotiana tabacum SHKB 1708        aacgtctgtaggctttctctaatttacccg-tatttttagtctgcatccttgttctcttg
Solanum tuberosum SHKB 1693        aacgtctgtaggcttatctaattacc-tgtactttttttagtatggattcttgttctcttg
Solanum lycopersicum SHKB 1683     aacgtctgtaggcttatctaatttacctgtacttctctagtatgaagtcttgtttgcttg
                                    *      *
```

```
Nicotiana sylvestris SHKA 1754  tcttttacttaaattatgaatcgctgtactaata----tttttagtttgga----a-tta
Petunia hybrida SHKA 1758       ttcttcaactaaactatgaatggatgtcttaata----tttactttttttt----t-t--
Capsicum annuum SHKA 1855       ttcttcacttatattatgaatgcttgtatgaaaa----a---ttatatttt------c--
Nicotiana tabacum SHKA 1792     --ctccgcttaaattatgaatgattgtatcagtg----ttcacattttttt------t--
Solanum tuberosum SHKA 1780      ttcttcacttatattatgaatgtttgtattaa-------aaattgtatttt------c--
Solanum lycopersicum SHKA 1803   ttcttcacttatattatgaatgtttgtattaaaa----aaaattgtatttt------c--
Capsicum annuum SHKB 1922       gtattcaaaacttatgtgtatgaggcctctggcc-----ttctgagtattc----gagat
Nicotiana sylvestris SHKB 1763  gtatccaataaactcagttaccagccatccgctcaaaacttatatgtggtctcggggcgt
Nicotiana tabacum SHKB 1768     gtatccaataaactcagttaccagccatccgctcaaaacttatatgtggtctcggggcgt
Solanum tuberosum SHKB 1728      gtatcc-aataacttat------------------------atatgtggcctctggccat
Solanum lycopersicum SHKB 1719   gtatcccaataacttat------------------------atatgtagcctctggccat
                                   *

Nicotiana sylvestris SHKA 1812  tttcttttaaatcttgaggatgtgttac--gtttaccttgtgttcatactggaagagtga
Petunia hybrida SHKA 1815       -caaagaccaatttggtggatatgttatgtttgcact--gtattcatactatagaagaga
Capsicum annuum SHKA 1897       -ttaaaacctatttggacgatatgatatgtttact--------------ga---aattgt
Nicotiana tabacum SHKA 1845     -taaaaaccatattggaggatatgttat--gtttg-cctatgtgcatacta---gaagag
Solanum tuberosum SHKA 1836     -ttaaatcctatttggaggatatatgttatgtttgcgctacgtgcatactg---aaattg
Solanum lycopersicum SHKA 1859  -ttaaatcctatttggaggatatatgttatgtttaccctacgtgcatactg---aaattg
Capsicum annuum SHKB 1972       tcactctactaattcggattcacgctatgtataatctataggaaaaaagc----------
Nicotiana sylvestris SHKB 1804  ttgagctgctatttggaaccaaagtt-tctaagt--------taaaacat----------
Nicotiana tabacum SHKB 1809     ttgagctgctatttggaaccaaagtt-tctaagt--------taaaacat----------
Solanum tuberosum SHKB 1774      ttgagctgctgtttggatccaatgtt-gaacatgttact---aataaaca----------
Solanum lycopersicum SHKB 1746   ccactgct-------------------aaac-acgtttt---aataaaca----------

Nicotiana sylvestris SHKA 1867  ----aaatgtatgagagcttg-atgtatcaaaacaaagtgtattagagtttgatgagtga
Petunia hybrida SHKA 1874       gtgttataatgtatgagagtt-tggtgaccaaggaataaagcaaggaagttactctaaaa
Capsicum annuum SHKA 1953       ttttgatgtaatgagagtttg-gtgggactaagcaataaagcaaggaagttactcta---
Nicotiana tabacum SHKA 1903     t-gctatgttgtatgagagtt-tggtgactaaggaataaagcaaggaagttactttgtct
Solanum tuberosum SHKA 1895      ttttaatgtaatgagagtttg-gtgagactaagcaataaagcaaggaagttactcttaaa
Solanum lycopersicum SHKA 1916   ttttaatgtaatgagagtttg-gtgagactaagcaataaagcaaggaagttactctat--
Capsicum annuum SHKB 2023       ---------attttgatcactattctcactacacattagtttaagtagtgttattagaag
Nicotiana sylvestris SHKB 1847  ---------gggcagagcaactttt------ccaaatgaggtaaataggtttcctg--cag
Nicotiana tabacum SHKB 1852     ---------gggcagagcaacttt------ccaaatgaggtaaataggtttcctg--cag
Solanum tuberosum SHKB 1815      ---------tggcagagcaacttg------cctat-gagg-taaatagatttctg--cag
Solanum lycopersicum SHKB 1789   ---------tgcctgagcaagttg------cctgatgaggtaaataggttttctg--cag
                                              **                                  *  *
```

```
Nicotiana sylvestris SHKA      tgagact---aagcaataaagcaaagaagttcctcttct---------------------
                 1903
Petunia hybrida SHKA           aaaaaaa---aaaaaaaa------------------------------------------
                 1889
Capsicum annuum SHKA           ------------------------------------------------------------
                 1953
Nicotiana tabacum SHKA         taaaaaa---aaaaaaaaaaaaaaaa----------------------------------
                 1926
Solanum tuberosum SHKA         aaaaaaa---aaaaaaaaaaaaaaaaaaaaaaaaaaa-----------------------
                 1929
Solanum lycopersicum SHKA      ------------------------------------------------------------
                 1916
Capsicum annuum SHKB           taaataacttagactatgaagggactgaataaataat-----------------------
                 2060
Nicotiana sylvestris SHKB      aaatcaatgggggaagtgaacagcaagtaaaattagtcgagttgtccggtagccattctg
                 1907
Nicotiana tabacum SHKB         aaatcaatgggggaagtgaacagcaagtaaaattagtcgagttgtccggtagccattctg
                 1912
Solanum tuberosum SHKB         agatcaaag-gggaaatgaacatcaa--gttattagtcgatttatccggtatctgttctg
                 1872
Solanum lycopersicum SHKB      tgatcaaag-gggaaatgaacatcaagtaaaattagtcgatttatccggcatctgttctg
                 1848
```

```
Nicotiana sylvestris SHKA      ------------------------------------------------------------
                 1903
Petunia hybrida SHKA           ------------------------------------------------------------
                 1889
Capsicum annuum SHKA           ------------------------------------------------------------
                 1953
Nicotiana tabacum SHKA         ------------------------------------------------------------
                 1926
Solanum tuberosum SHKA         ------------------------------------------------------------
                 1929
Solanum lycopersicum SHKA      ------------------------------------------------------------
                 1916
Capsicum annuum SHKB           ------------------------------------------------------------
                 2060
Nicotiana sylvestris SHKB      tttattaaaattttaaaatttagtttggactgtaagtttagggtataagtgggttcagtt
                 1967
Nicotiana tabacum SHKB         tttattaaaattttaaaatttagtttggactgtaagtttagggtataagtgggttcagta
                 1972
Solanum tuberosum SHKB         tttatttta-------------gtttggactgtaagtctagggtataaggatgttctttt
                 1919
Solanum lycopersicum SHKB      tttatttta-------------gtttggactgtaagtctaggttttaagggtgttcagtt
                 1895
```

```
Nicotiana sylvestris SHKA      ------------------------------------------------------------
                 1903
Petunia hybrida SHKA           ------------------------------------------------------------
                 1889
Capsicum annuum SHKA           ------------------------------------------------------------
                 1953
Nicotiana tabacum SHKA         ------------------------------------------------------------
                 1926
Solanum tuberosum SHKA         ------------------------------------------------------------
                 1929
Solanum lycopersicum SHKA      ------------------------------------------------------------
                 1916
Capsicum annuum SHKB           ------------------------------------------------------------
                 2060
Nicotiana sylvestris SHKB      -ctctcctttttcttgatgtatggattttattttgagatactggttttgcagtggag---t
                 2023
Nicotiana tabacum SHKB         tttctcctttttcttgatgtatggattttattttgagatactggttttgcagtggag---t
                 2029
Solanum tuberosum SHKB         -----tctttccttgatgtatgaatgatactttgagatactggttttgctgttttagtct
                 1974
Solanum lycopersicum SHKB      -----tttctt---------------------tgggatactggttttgctgttttagtct
                 1929
```

```
Nicotiana sylvestris SHKA      ------------------------------------------------------------
                 1903
Petunia hybrida SHKA           ------------------------------------------------------------
                 1889
Capsicum annuum SHKA           ------------------------------------------------------------
                 1953
Nicotiana tabacum SHKA         ------------------------------------------------------------
                 1926
Solanum tuberosum SHKA         ------------------------------------------------------------
                 1929
Solanum lycopersicum SHKA      ------------------------------------------------------------
                 1916
Capsicum annuum SHKB           ------------------------------------------------------------
                 2060
Nicotiana sylvestris SHKB      caagtgtttgcctaccttcttgc-------------------------------------
                 2046
Nicotiana tabacum SHKB         caagtgtttgcctaccttcttgccatttttgcttattttacagctattagattgtatttt
                 2089
Solanum tuberosum SHKB         aatgtgtttgcctaccttgtc---------------------------------------
                 1995
Solanum lycopersicum SHKB      aatgtgtttgccta----------------------------------------------
                 1943
```

```
Nicotiana sylvestris SHKA     --------------------------------------------------------
Petunia hybrida SHKA          --------------------------------------------------------
Capsicum annuum SHKA          --------------------------------------------------------
Nicotiana tabacum SHKA        --------------------------------------------------------
Solanum tuberosum SHKA        --------------------------------------------------------
Solanum lycopersicum SHKA     --------------------------------------------------------
Capsicum annuum SHKB          --------------------------------------------------------
Nicotiana sylvestris SHKB     --------------------------------------------------------
Nicotiana tabacum SHKB        cataatttgtttcctataatggcattttactggatagaagtgcataatctgcagag
             2145
Solanum tuberosum SHKB        --------------------------------------------------------
Solanum lycopersicum SHKB     --------------------------------------------------------
```

Figure S2. CLUSTAL Omega multiple sequence alignment of the two DAHP synthase isogene families from selected members of the *Solanaceae*. *SHKA* accession numbers are XM_009795179 for *Nicotiana sylvestris*, JQ955569 for *Petunia hybrida*, XM_016713717 for *Capsicum annuum*, NM_001325203 for *Nicotiana tabacum*, NM_001288432 for *Solanum tuberosum* and NM_001247489 for *Solanum lycopersicum*. *SHKB* accession numbers are XM_016694065 for *Capsicum annuum*, XM_009771454 for *Nicotiana sylvestris*, XM_016642994 for *Nicotiana tabacum*, NM_001288470 for *Solanum tuberosum* and NM_001247486 for *Solanum lycopersicum*. Primers used to amplify cDNA from *Nicotiana plumbaginifolia* are shaded in red (SHKA) or light blue (SHKB).

```
DAHPs1      ------------------------------------------------------------  0
DAHPs2      GTGCTGAGAGTTTTAAGGAATTTAATGCTAATAATATTCGTGATACTTTTAGGATTCTTC  60


DAHPs1      ------------------------------------------------------------  0
DAHPs2      TTCAGATGAGTGTTGTTCTTATGTTTGGTGGTCAAGTTCCTGTGATCAAGGTTGGAAGAA  120


DAHPs1      ------------------------------------------AGAAAGATGGAGTAAAGC  18
DAHPs2      TGGCGGGTCAGTTTGCGAAACCAAGGTCAGATCCGTTTGAGGAGATTAATGGGGTGAAGC  180
                                                      ***   **** ** ****

DAHPs1      TGCCAAGTTACAGGGGAGATAATGTGAATGGAGATGCATTTGATGAGAAATCCAGAACTC  78
DAHPs2      TGCCAAGTTACAAGGGTGATAACATTAATGGTGATACATTTGATGAGAAGTCAAGGATTC  240
            ************ *** *****   * ***** *** ************* ** ** * **

DAHPs1      CGGACCCTCAAAGGATGATCAGGGCCTATTGTCAATCTGCAGCTACTTTGAATCTCTTGA  138
DAHPs2      CAGATCCTCATAGGCTTATTAGGGCTTACATGCAATCTGCTGCCACTCTTAACCTTCTTA  300
            * ** ***** *** * ** ***** **     ******** ** *** * ** **  * *

DAHPs1      GGGCCTTTGCTAAAGGAGGATATGCTGCCATGCAGAGGATCAACCAATGGAACTTAGATT  198
DAHPs2      GAGCTTTTGCTACCGGAGGTTATGCTGCAATGCAGAGGGTCACCGAATGGAATCTTGATT  360
            * ** *******  ***** ******** ********* *** * *******  * ****

DAHPs1      TTACAGAGCACAGTGAGCAGGGTGATCGGTATCGTGAATTAGCTCATAGAGTGGATGAGG  258
DAHPs2      TTGTGGAGAACAGTGAGCAAGGAAATAGGTATCAAGAACTAGCTCACAGGGTTGATGAAG  420
            **   *** ********** **  ** ******  *** ******* ** ** ***** *

DAHPs1      CCCTTGGTTTCATGGCTGCTGCTGGACTTACAACGGATCATCCTATCATGAAAACAACTG  318
DAHPs2      CCTTGGGATTCATGGCTGCTGCTGGACTTACAGTTGACCACCCTATCATGGCGACAACTG  480
            ** * ** ************************    ** ** *********   *******

DAHPs1      AGTTTTGGACGTCTCACGAGTGCTTACTTTTGCCGTATGAGCAATCACTTACAAGATTGG  378
DAHPs2      ATTTCTGGACATCCCATGAGTGCTTGCTTCTTCCTTATGAACAAGCACTTACAAGGGAGG  540
            * ** ***** ** ** ******** *** * ** ***** *** **********   **

DAHPs1      ATTCAACTTCTGGCCTTTACTATGATTGCTCTGCCCATTTTCTTTGGGCTGGGGAGAGAA  438
DAHPs2      ATTCAACTTCTGGTCTTTTCTATGATTGTTCCGCTCACATGATTTGGGTTGGTGAACGAA  600
            ************* **** ********* ** **  *   ****** *** **   ***

DAHPs1      CCAGACAATTGGATGGTGCCCATGTTGAGTTCTTGAGAGGAATTGCCAACCCCCTTGGGA  498
DAHPs2      CCAGGCAACTTGACGGTGCT----------------------------------------  620
            **** *** * ** *****

DAHPs1      TAAAGGTGAGTGACAAGATGGATCCAAATGAGTTAGTTAAGCTCATTGACATTCTGAACC  558
DAHPs2      --------------------CATGCAAATGAGCTAGTTAAACTCATTGACATCCTGAACC  660
                                 ** ******** ******* *********** *******

DAHPs1      CTAACAACAAACCAGGAAGGATTACAATAATTACCAGAATGGGAGCAGAGAACATGAGGG  618
DAHPs2      CAACCAATAAGCCCGGAAGAATTACTGTAATTGTGAGAATGGGTGCTGATAATATGAGAG  720
            * * *** ** ** ***** *****     *****   ******** ** ** ** ***** *

DAHPs1      TTAAGCTTCCTCATTTAATAAGGGCAGTGAGAAGAGCTGGGCAAATTGTTACGTGGGTAT  678
DAHPs2      TGAAGCTTTGCCACTTGATCAGGGCTGTTCGAGGAGCTGGACAGATTGTTACCTGGGTTT  780
            * ******    ** ** ** ***** **  ** ******* ** ******** ***** *

DAHPs1      CTGATCCTATGCATGGAAATACCATTAAAGCACCTTGTGGTCTGAAAACTCGACCTTTTG  738
DAHPs2      GTGACCCGATGCACGGCAACACCATAAAGGCACCATGTGGACTCAAAACCC---------  831
             *** ** ***** ** ** ***** ** ***** ***** ***** ** ***** *

DAHPs1      ATGCCATCAGGGCTGAAGTGAGAGCATTCTTTGACGTGCATGAGCAAGAAGGAAGCCATC  798
DAHPs2      ------------------------------------------------------------  831


DAHPs1      CAGGA          803
DAHPs2      -----          831
```

Figure S3. Alignment of partial cDNA sequences of the two DAHP synthase isoforms from *Nicotiana plumbaginifolia*. Primers used for real-time PCR analysis are shaded in red (SHKA) or light blue (SHKB). Sequences were deposited in GenBank under accession numbers MT811773 and MT811774, respectively.

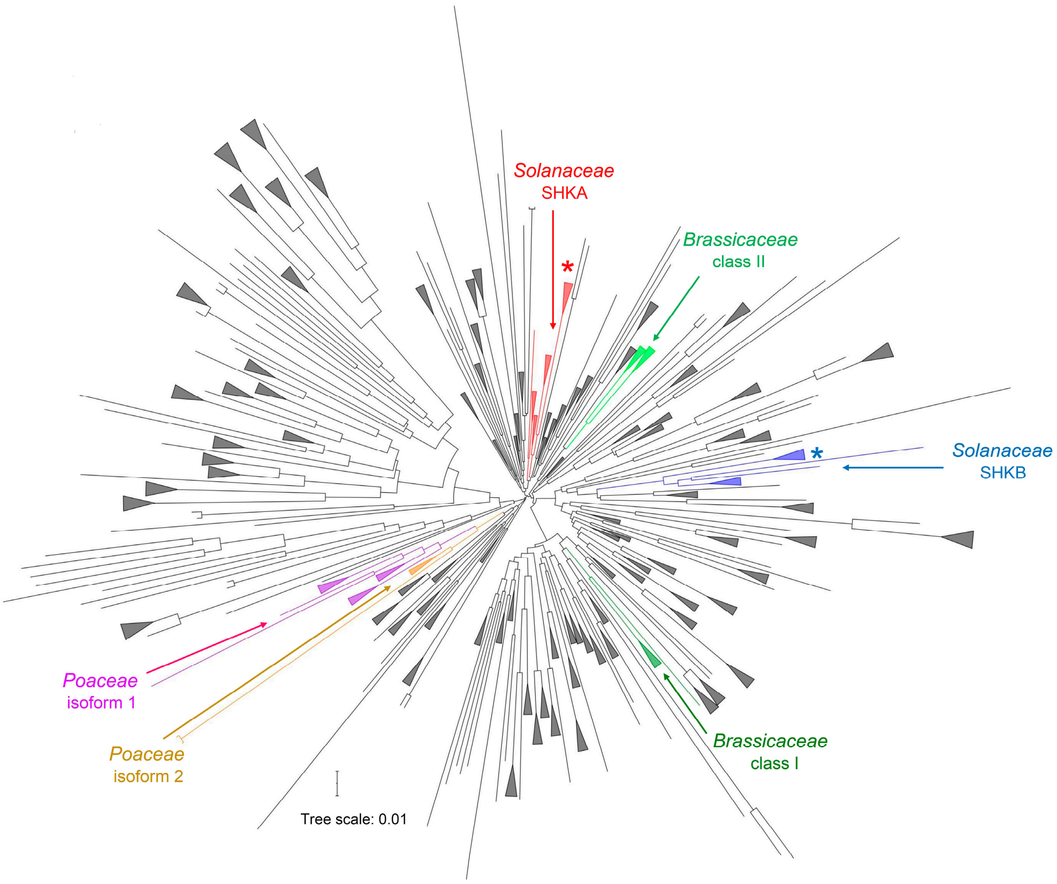

Figure S4. Phylogenetic relationships of wild tobacco DAHP synthase isoforms with the enzymes from other plants. Sequences of plant DAHP synthases were retrieved from the NCBI non-redundant protein sequences database by similarity with *N. tabacum* SHKA (accession number NP_001312132.1) using standard protein BLAST (https://blast.ncbi.nlm.nih.gov/). Results were filtered by coverage (>80% of the query) and identity (>65%). The 741 sequences obtained were aligned with Clustal Omega (https://www.ebi.ac.uk/Tools/msa/clustalo/), and the resulting Neighbour-joining tree was visualized with iTOL (https://itol.embl.de/). Results showed distinct clades for multiple enzyme forms in the members of *Solanaceae*, *Brassicacee* and *Poaceae*. While in the last case both clades derive from the same node, for the former two families they clustered well apart from each other, suggesting that gene duplication occurred before evolutionary divergence. The position of *N. plumbaginifolia* DAHP synthases is indicated by red and blue asterisks, respectively.
